# Small Sized Yet Powerful: Nuclear Distribution C Proteins in Plants

**DOI:** 10.3390/plants13010119

**Published:** 2023-12-31

**Authors:** Valya Vassileva, Mariyana Georgieva, Dimitar Todorov, Kiril Mishev

**Affiliations:** Department of Molecular Biology and Genetics, Laboratory of Regulation of Gene Expression, Institute of Plant Physiology and Genetics, Bulgarian Academy of Sciences, 1113 Sofia, Bulgaria; stamova@bio21.bas.bg (M.G.); d-todorov@bio21.bas.bg (D.T.)

**Keywords:** *BOB1*, chaperone activity, *NMig1*, *Nud* family, plant development, stress response

## Abstract

The family of Nuclear Distribution C (NudC) proteins plays a pivotal and evolutionarily conserved role in all eukaryotes. In animal systems, these proteins influence vital cellular processes like cell division, protein folding, nuclear migration and positioning, intracellular transport, and stress response. This review synthesizes past and current research on NudC family members, focusing on their growing importance in plants and intricate contributions to plant growth, development, and stress tolerance. Leveraging information from available genomic databases, we conducted a thorough characterization of NudC family members, utilizing phylogenetic analysis and assessing gene structure, motif organization, and conserved protein domains. Our spotlight on two Arabidopsis *NudC* genes, *BOB1* and *NMig1*, underscores their indispensable roles in embryogenesis and postembryonic development, stress responses, and tolerance mechanisms. Emphasizing the chaperone activity of plant NudC family members, crucial for mitigating stress effects and enhancing plant resilience, we highlight their potential as valuable targets for enhancing crop performance. Moreover, the structural and functional conservation of NudC proteins across species suggests their potential applications in medical research, particularly in functions related to cell division, microtubule regulation, and associated pathways. Finally, we outline future research avenues centering on the exploration of under investigated functions of NudC proteins in plants.

## 1. Nuclear Distribution Genes and Their Functional Significance across Eukaryotes

**Figure 1:**
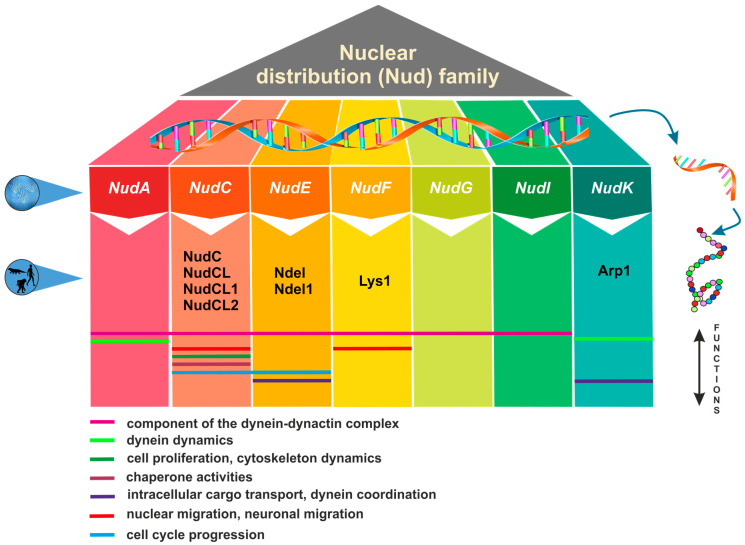
Overview of nuclear distribution (Nud) family members and their cellular functions.

The nuclear distribution (*Nud*) genes encode evolutionary conserved proteins, which play an important role in various cellular processes. A notable example of *Nud* gene function, which inspired their name, is nuclear migration, a precisely regulated process that holds particular importance in highly elongated cells [1,2]. *Nud* genes were originally discovered in the filamentous fungus *Aspergillus nidulans* [3,4,5,6]. Xiang et al. [3] have identified four *Nud* genes, namely *NudA*, *NudC*, *NudF*, and *NudG*, encoding components of the cytoplasmic dynein–dynactin complex. NudC proteins are known for their compact structure and versatile functions, encompassing the facilitation of cell division, the regulation of gene expression, and contribution to various essential biological processes (Figure 1, Table S1). Deletions in the *NudC* gene lead to a more severe phenotype compared to other nuclear distribution mutants, and can result in lethality [4]. *NudC* acts in concert with the molecular motor dynein and other *Nud* genes to regulate dynein-mediated processes, including vesicle transport in neurons [7,8], Golgi apparatus positioning, lysosomes and vesicles transport [9], kinetochore localization, spindle organization [10], phagosome movement [3], nuclear transport, and membrane organelle organization [7]. The *NudA*, *NudI*, and *NudG* genes encode the heavy, intermediate, and light chains of cytoplasmic dynein, respectively [5,11,12]. The NudF protein is required for nuclear migration through the fungal mycelium, and interacts with microtubule-related proteins, such as α-tubulin and dynein [13,14,15]. The *NudF* gene encodes a protein that exhibits a 42% sequence similarity with the human *Lis1* gene, which is associated with Miller–Dieker syndrome. The hemizygous deletion or mutation of the *Lis1* gene results in type I lissencephaly, a condition that obstructs proper neuronal migration during brain development and leads to a smooth brain surface and disorganized cortical layering [16,17].

*Lis1* plays a role in mitosis both through direct mechanisms [18,19,20] as well as indirectly by impeding the movement of nuclei in radial glial progenitor cells towards the ventricular surface [21]. The *NudE* gene was subsequently identified as a multicopy suppressor of the *NudF* phenotype, demonstrating its capacity to rescue the aberrant characteristics associated with *nudF* mutations through the introduction of multiple *NudE* copies [22]. Furthermore, *NudE* possesses two homologs, *Ndel* and *Ndel1*, which play a role in recruiting dynein to mitotic kinetochores, facilitating the progression of mitosis [23,24]. Mutant mice lacking both *Ndel* and *Ndel1* genes can exhibit varying neurological conditions, contingent upon the gene dosage. In particular, they may develop microcephaly due to mitotic abnormalities [25] or lissencephaly, which aligns with their role in the cytoplasmic dynein pathway [26]. Noteworthy, mutations in human *NudE* can lead to the co-occurrence of microcephaly and lissencephaly [27]. It has been shown that Lis1, in conjunction with NudE, orchestrates the assembly of an intricate molecular complex involving cytoplasmic dynein [28]. Within this assembly, Lis1, under the guidance of NudE, transforms dynein into a state of sustained force production, which proves to be a vital factor in facilitating the transport of heavy-load structures, including cell nuclei. Interestingly, *NudE* appears to be intricately connected within the same genetic pathway as *NudF* and *NudA*. The phenotypes observed in *nudE* homozygous mutants closely resemble those of *nudF* and *nudA* heterozygous mutants, suggesting that *NudF*, *NudE*, and *NudA* operate within the same functional pathways [29]. A crucial element in the complex network of Nud proteins is the actin-related protein Arp1, encoded by the *NudK* gene. Arp1 assumes a pivotal function in the dynactin complex [30] by activating dynein and consequently enhancing the intracellular cargo transport [31]. NudC also regulates the level of NudF protein in *A. nidulans* [6]. A *nudC* mutation leads to a significant decrease in NudF, primarily at the restrictive temperature. On the other hand, additional copies of the *NudF* gene can effectively complement the temperature-sensitive phenotype of *nudC* mutants [5,32,33].

These findings provide valuable insights into the intricate network of Nud proteins and underscore their vital roles in various cellular processes (Figure 1). The involvement of the *Nud* gene family in nuclear migration and the cytoplasmic dynein–dynactin complex and their connections to human conditions, like Miller–Dieker syndrome and lissencephaly, emphasize their dual significance in both normal cellular functions and the pathophysiology of diseases.

## 2. Structural and Functional Features of NudC Proteins

**Figure 2:**
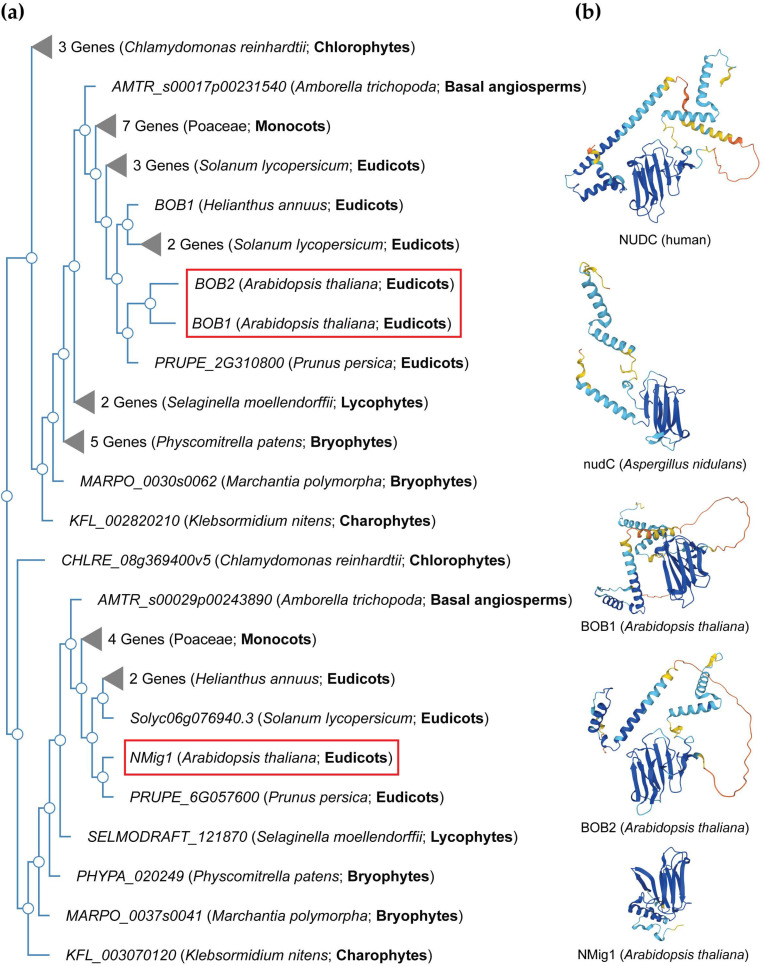
NudC protein conservation across eukaryotes. (**a**) Simplified phylogenetic tree showing the evolutionary relationships of plant and algal NudC proteins. The phylogenetic tree was generated using PhyloGenes (https://phylogenes.arabidopsis.org, accessed on 10 November 2023) with the following selected species (retrieved AtBOB1 gene homologs): *Arabidopsis thaliana* (3), *Solanum lycopersicum* (6), *Prunus persica* (2), *Helianthus annuus* (3), *Hordeum vulgare* (4), *Oryza sativa* (3), *Zea mays* (4), *Amborella trichopoda* (2), *Selaginella moellendorffii* (3), *Physcomitrella patens* (6), *Marchantia polymorpha* (2), *Klebsormidium nitens* (2), and *Chlamydomonas reinhardtii* (4). Details are provided in Table S1. (**b**) Predicted protein structures of NudC family members in human, fungi, and Arabidopsis. The protein structures were generated and visualized with the help of the AlphaFold algorithm of EMBL-EBI (https://alphafold.ebi.ac.uk, accessed on 10 November 2023). Red rectangles outline the Arabidopsis *NudC* genes.

NudC proteins display a remarkable degree of conservation across a broad spectrum of organisms, spanning from fungi and plants to animals [34] (Figure 2, Table S1). The *NudC* homologous genes or proteins are found in a number of higher eukaryotes, including *Caenorhabditis elegans* [35], *Drosophila melanogaster* [36], newts [37], plants [38,39,40], and mammals [41]. The NudC family consists of four proteins: NudC [42,43,44], NudC-like (NudCL) also known as NudC domain-containing protein 3 (NUDCD3) [45,46], NudC-like 2 (NudCL2/NUDCD2) [47], and the more distant NudC domain-containing 1 (NudCD1) also known as CML66 (Chronic Myeloid Leukemia 66) (Table S1).

NudC proteins typically exhibit a conserved domain architecture and contain specific motifs that are crucial for their functional roles in intracellular transport, nuclear positioning, cell cycle progression and stress responses (Figure 2b). They include a coiled-coil region at the N-terminus serving as the dimerization module, and also possess a central globular domain, a CS domain (a domain shared by CHORD-containing proteins and SGT1, PF04969) resembling p23 and other small heat shock proteins (sHSPs), and two conserved α-helices downstream [48,49,50]. This architecture is maintained across various species, albeit with some variations in the N-terminus. Compared to fungi, higher eukaryotes typically possess an extended N-terminal segment. In vertebrates, the most substantial amino acid conservation is observed in a short N-terminal segment and the C-terminal α-helices [50]. It is essential to note that NudC molecules operate as dimers, and their biological roles may depend on this dimeric state. Common structural features between NudC and NudCL imply a potential functional overlap, despite the relatively low amino acid sequence similarity. NudCL is unique to the animal kingdom, whereas NudCL2 is found across all eukaryotes, distinguishing itself by the absence of an N-terminal extension and featuring a distinct C-terminal fragment [50].

The architectural variations seen in NudC protein family across different species (Figure 2b) present an intriguing spectrum of structural diversity. This diversity highlights the remarkable adaptability of the NudC protein family to different biological contexts, providing compelling insights into the potential existence of variable functional roles played by the NudC protein family in various organisms. Understanding their structural adaptability contributes to unraveling the complexities of their functions, positioning the NudC protein family as dynamic contributors to biological processes across the evolutionary landscape.

The expression of NudC is markedly associated with the rate of proliferation in diverse cell types and tissues. The human NudC homolog displays robust expression in actively proliferating cells [51] with a central role in spindle formation during mitosis [12]. Depletion of *NudC* results in the presence of multiple spindles during metaphase and lagging chromosomes during anaphase [52]. Remarkably, both the depletion and overexpression of *NudC* components can induce cytokinesis defects in mammalian cells [52,53]. NudC deacetylation is an important player in the regulation and coordination of mitotic progression [54]. Additionally, NudC is a pivotal regulator of actin polymerization and depolymerization [55]. In *A. nidulauns*, the *NudC* gene is involved in the movement of nuclei during the asexual reproductive cycle and deposition of the cell wall, colony growth, and overall viability [4,6]. NudC homologs from *D. melanogaster*, *C. elegans*, *Arabidopsis thaliana*, and mammals are capable of complementing the *nudC3* mutation in *A. nidulans*, restoring the normal movement of nuclei and promoting colony growth [35,36,43,56]. These findings strongly suggest that the role of NudC in nucleus movement is preserved throughout the evolution of eukaryotes.

As already noted, all NudC members share a common CS domain, which is typical for proteins with chaperone or co-chaperone activities [50,57]. The CS domain in the NudC family mirrors the molecular architecture of sHSPs, such as HSP20/α-crystallin and p23 proteins [49]. It is important to mention that HSP20 and p23 serve as central structures responsible for interacting with HSP90 and specific client proteins [49,57,58,59]. This implies that proteins containing these structures are instrumental in recruiting HSPs to multiprotein complexes [57]. Taipale et al. [60] has shown that co-chaperones of the NudC family specifically associate with protein partners that contain β-propeller domains. Experimental evidence supports both the HSP90 co-chaperone and inherent chaperone functions of the NudC family. For instance, the microtubule-associated *C. elegans* NudC homolog, *NUD-1*, has demonstrated in vitro chaperone activity by preventing the heat-induced aggregation and precipitation of citrate synthase and luciferase [61]. Likewise, human NudC stabilizes Lis1 through HSP90-mediated pathways and exhibits intrinsic chaperone activity in vitro, inhibiting the aggregation of citrate synthase [47,62]. Utilizing a combination of Nuclear Magnetic Resonance, crystallography, and modeling techniques, it has been demonstrated that NudC interacts with HSP40, HSP90, and the client receptor [63]. NudC facilitates the transfer of clients to HSP90 by recruiting substrates bound to HSP40 while excluding HSP70, ultimately expediting client activation [63,64,65].

Therefore, the NudC family members collectively play a vital role in cell functions, involving microtubule regulation, chaperone activities, and diverse protein interactions. Their substantial conservation and occurrence in all tissues and cells during development and maturity underscore their significance in upholding cell integrity and various processes, highlighting their adaptability across species.

## 3. NudC Proteins in Plants

Plant biologists have increasingly recognized the importance of *NudC* family members due to their involvement in fundamental cellular processes critical for plant growth, development, and response to environmental challenges (Table S1). Nevertheless, to date, very few studies have explored NudC members in plants and provided information for their roles in the plant kingdom [38,39,40,66,67,68,69], and a substantial amount of additional research is necessary to fully elucidate their functions.

### 3.1. Structural Features of Plant NudC Members

**Figure 3:**
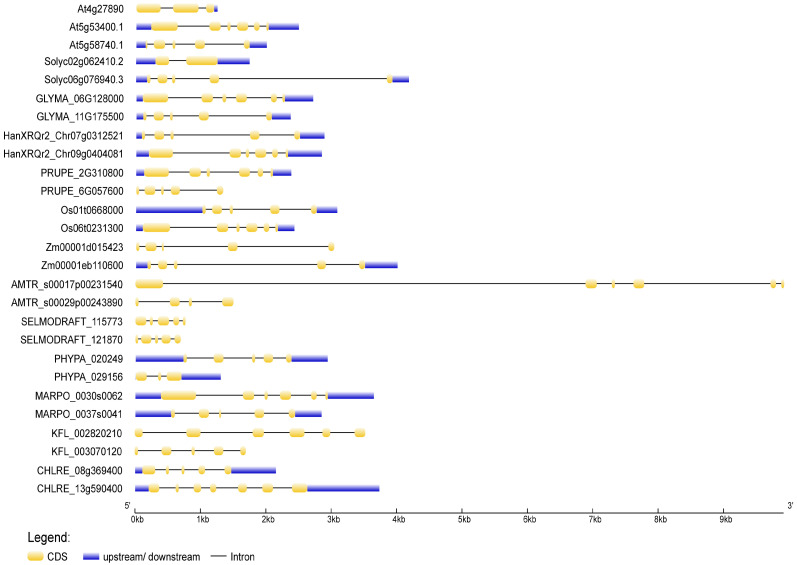
Exon–intron structures of selected plant and algal *NudC* genes. Untranslated regions are depicted by blue boxes, coding regions by yellow boxes, and introns by lines. The gene lengths can be estimated using the scale provided at the bottom. The exon–intron structures were generated and visualized using the Gene Structure Display Server (http://gsds.gao-lab.org, accessed on 11 November 2023).

To deduce the evolutionary history of the *NudC* gene family, we traced the dynamics of intron–exon organization in selected *NudC* genes across a spectrum of plant species. The genomic and coding sequences (CDS) of NudC members were acquired from the Ensembl Plants (https://plants.ensembl.org, accessed on 10 November 2023) and the National Center for Biotechnology Information (NCBI, https://www.ncbi.nlm.nih.gov, accessed on 10 November 2023) databases. The analysis was conducted using the Gene Structure Display Server (GSDS) [70] available at http://gsds.gao-lab.org/, accessed on 10 November 2023. The positions of introns and exons in *NudC* genes were ascertained through the identification of gaps during the alignment of full-length cDNA transcripts with genomic sequences (Figure 3). Our investigation commenced with single-cell green algae *Chlamydomonas reinhardtii*, the charophytic algae *Klebsormidium nitens*, and the bryophytes *Marchantia polymorpha* and *Physcomitrella patens*. Subsequently, we explored the lycophyte *Selaginella moellendorffii*, the evergreen shrub *Amborella trichopoda*, and advancing up the evolutionary scale, extended to monocot and dicot higher plants, such as *Zea mays*, *Oryza sativa*, the deciduous tree *Prunus persica*, the annual forb *Helianthus annuus*, the legume *Glycine max*, the cultivated tomato *Solanum lycopersicum*, and the widely used plant model species *Arabidopsis thaliana*.

In the Arabidopsis genome, three *NudC* genes have been identified. One is designated as *BOBBER1* (*BOB1*), which has a paralog named *BOBBER2* (*BOB2*) [38,39,67]. The third member is referred to as *NUCLEAR MIGRATION 1* (*NMig1*), and displays a more distant relationship to the other two NudC members (Figure 2a). It also has a distinct architectural arrangement and a shorter protein sequence (Figure 3, Figure S1). Given this variation, our analysis focused on two NudC members from each species under examination—one related to BOB1 and the other to NMig1.

In general, a considerable structural diversity was observed in the number, size, and distribution of exons and introns among the *NudC* members (Figure 3). The number of exons ranged from one to nine, as detailed in Table S1. In *Z. mays*, both investigated *NudC* genes exhibited a tendency for extended introns and shorter exons. The second intron of the *BOB1*-related *A. trichopoda* gene, which stood out as the longest, resulted in a genomic DNA sequence spanning 9918 kb. Conversely, in *S. moellendorffii*, *A. thaliana*, and the *BOB1*-related *S. lycopersicum* gene, the distribution leans towards more evenly sized introns and exons. Not all *NudC* genes comprised 5′ and 3′ untranslated regions (UTRs). The overall gene exon–intron organization indicated a lack of apparent correlation with phylogeny, suggesting that *NudC* genes may have undergone evolutionary changes such as gene duplication, exon shuffling, or other mechanisms influencing gene structure. This structural diversity underscores the evolutionary dynamics shaping the structural features of this gene family in different biological contexts.

**Figure 4:**
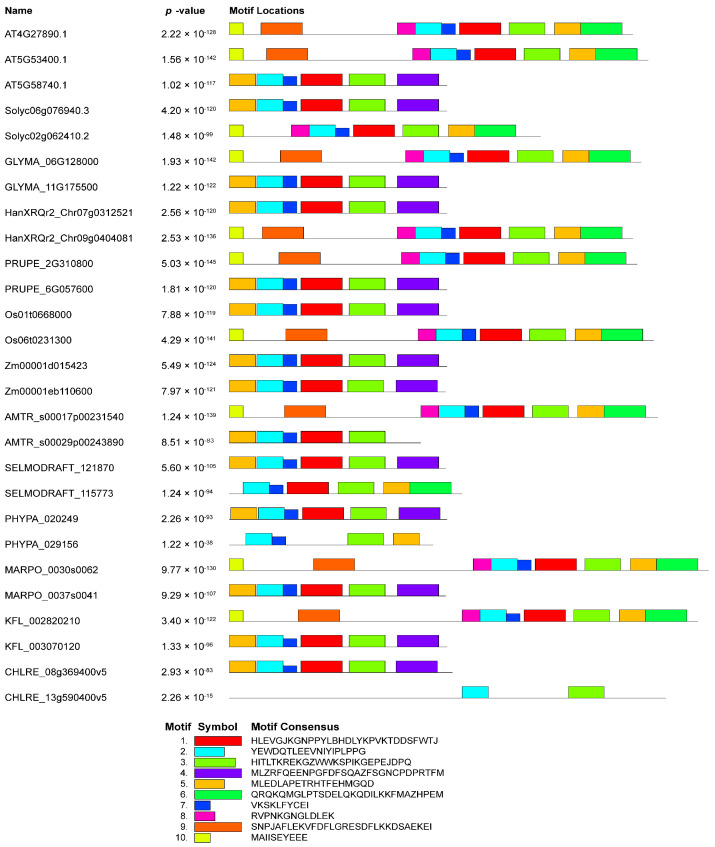
Motif analysis of selected plant and algal NudC genes. Numbered colored boxes (1–10) represent distinct patterns with corresponding specific motifs. The motifs were generated and visualized using the online tool Multiple Em for Motif Elicitation (https://meme-suite.org/meme/tools/meme, accessed on 12 November 2023).

Furthermore, we employed the online tool Multiple Em for Motif Elicitation (MEME Suite version 5.5.5) (https://meme-suite.org/meme/tools/meme, accessed on 10 November 2023) to identify conserved motifs within NudC protein sequences [71]. The details of 10 motifs are provided in Figure 4. The number of preserved motifs ranged from 2 to 9 across different NudC members. Particularly noteworthy is the uniformity among NMig-related protein members, demonstrating identical types and numbers of motifs with consistent distribution. Specifically, these proteins shared motifs 1, 2, 4, 5, 6, and 7. The only exception is observed in the *A. trichopoda* protein AMTR_s00029p00243890, which lacks motif 4. Certain motifs were restricted to the BOB1 group, such as motifs 3, 8, 9, and 10, while motif 4 was uniquely present in NMig-related protein members. Among the 10 distinct conserved motifs, motif 2 was universally present in all studied proteins, whereas motif 5 and 7 were absent only in the *C. reinhardtii* CHLRE_13g590400v5. This particular protein exhibits a relatively simple motif composition, featuring only motifs 2 and 3. Therefore, our analysis has shown a noteworthy paradox: despite the apparent structural diversity observed within this gene family, there exists a remarkable commonality in the presence of conserved motifs, which could be predominantly tethered to the phylogenetic relationships among NudC family members.

Additionally, we utilized the web-based Batch CD-Search tool from NCBI (https://www.ncbi.nlm.nih.gov/Structure/bwrpsb/bwrpsb.cgi, accessed on 12 November 2023) [72] to annotate the positions of conserved domains within the protein sequence of selected plant NudC homologs (Figure S1). Two highly conserved domains were identified: the CS domain and the p23_NUDC-like domain (cd06467, NCBI). All plant NudC members shared both conserved domains, with a high overlap in their protein sequences. Within the extended proteins of BOB1-like members, the conserved domains exhibit a tendency to reside towards the C-terminus. By contrast, in the NMig1-like group they encompass nearly all NudC proteins. The conservation of both domains is observed not only in plant species but also in other eukaryotes (Table S1).

In conclusion, our thorough analysis of the NudC gene structure across a diverse range of plant species has revealed both variability and common features within this gene family across various biological contexts.

### 3.2. Developmental Role of Plant NudC Proteins

The *A. thaliana* gene *BOB1* encodes a small 34.5 kDa protein featuring the NudC domain, which specifies a non-canonical sHSP with versatile functions, contributing to developmental processes and responses to elevated temperatures [38,39,67]. The other two NudC genes in the Arabidopsis genome, *BOB2* and *NMig1*, have also been identified as CS domain-containing NudC family members (Figure S1).

The importance of the *BOB1* gene becomes evident in its contribution to the precise partitioning and patterning of the apical domain in Arabidopsis embryos [38]. The loss of *BOB1* results in embryo lethality, linked to developmental arrest at the globular stage without progressing to the transition stage. The *bob1* null mutants exhibit an expansion of meristematic identity into the region that would typically give rise to cotyledons, leading to the failure of cotyledon formation. These developmental anomalies coincide with atypical gene expression patterns, particularly the overexpression of the meristem-specific *SHOOTMERISTEMLESS* (*STM*) gene in the upper half of the embryo. At the same time, the expected expression of the *AINTEGUMENTA* (*ANT*) transcripts in cotyledons is absent. It is noteworthy that *bob1* mutant embryos do not establish auxin gradients [38], which suggests a potential interaction between BOB1 and the auxin-mediated developmental processes.

These findings underscore the critical role of this *NudC* gene in orchestrating key aspects of embryonic development and its potential involvement in regulating auxin-related developmental pathways.

The importance of *NudC* genes extend beyond embryonic development to postembryonic phases. The hypomorphic *bob1-3* allele introduces a broad spectrum of developmental abnormalities, including shorter roots, smaller serrated leaves, stunted branched inflorescences, and irregularities in inflorescence and floral meristem formation, leading to pin-formed meristems and abnormal floral organ numbers [39]. It is noteworthy to mention that many of these phenotypic anomalies closely correspond to those seen in mutants associated with impaired auxin signaling or transport. Moreover, the formation of serrations on the edges of *bob1-3* leaves is contingent upon the activity of the PIN1 protein, underscoring the influence of BOB1 on this central player in the regulation of auxin efflux [38,73]. These observations provide compelling evidence for the critical involvement of BOB1 in shaping the auxin-mediated developmental landscape.

In addition, the genetic interplay of *BOB1* with *AS1* and *AS2* provides valuable insights into a developmental pathway that relies on the function of this *NudC* gene. *AS1* and *AS2* serve as transcriptional regulators, contributing to the establishment of meristem boundaries by suppressing *KNOX* gene expression and reinforcing adaxial polarity during leaf development [74,75,76]. An allelic variant of *BOB1*, known as *eal-1* and sharing the same mutation as *bob1-3*, offers a unique perspective in this context. Remarkably, *eal-1* represents the sole viable allele of *BOB1* with well-documented phenotypes [39]. When combined with *as1* and *as2* mutations, *eal-1* demonstrates leaf morphology characterized by abaxialized filamentous structures, along with an upregulation of *KNOTTED-like* homeobox (*KNOX*) and *ETTIN* (*ETT*)/*AUXIN RESPONSE FACTOR3* (*ARF3*) genes. *ETT* plays a role in enhancing abaxial identity and is directly regulated by the AS1–AS2 complex [75]. The observed polarity abnormalities in *eal-1*; *as2* plants are mitigated in an *ett* genetic background, suggesting that *ETT* acts downstream of *BOB1*, *AS1*, and AS2 [66]. These findings illuminate the intricate interactions among BOB1, AS1, and AS2 within the context of leaf development.

Recent studies conducted by Velinov et al. [40,69] have advanced the understanding of the functions of NudC members in postembryonic root development in Arabidopsis. These studies have provided substantial insights into an uncharacterized Arabidopsis homolog of *NudC* genes, designated as *NMig1* (*Nuclear Migration 1*). In-depth exploration of *NMig1* has revealed that its constitutive overexpression leads to enhanced root growth and increased lateral root development under a range of conditions, including both optimal and abiotic stress scenarios [40]. Conversely, *nudC* mutant plants have exhibited a noticeable reduction in primary root growth and lateral root development when compared to the wild-type Col-0 [69]. Furthermore, GUS expression analysis has revealed prominent expression of *NMig1* in the root meristem, emphasizing its importance for root development. This expression has been detected at various stages of lateral root primordia formation. During the initial phases, *NMig1* expression encompassed the entire lateral root primordia, while as development progressed, the highest expression levels became concentrated in the central, actively dividing cells that constitute the core of these primordia.

These findings underscore the fundamental importance of *NudC* genes in leaf development and their role in guiding the initial phases of lateral root formation and ultimately determining the final root architecture. Furthermore, these investigations emphasize the promising potential of *NudC* family members as valuable targets for crop improvement, especially when focused on optimizing plant architecture to enhance crop performance.

### 3.3. Unveiling the Role of NudC Proteins in Plant Stress Response and Resilience

The structural similarities between the NudC domain and α-crystallin domain (ACD)/p23 proteins [49] suggest the potential for shared functions with ACD-containing sHSPs [77,78]. The ACD, initially identified in the chaperone of the vertebrate eye lens, spans approximately 80–100 amino acids and is primarily located in the C-terminal domain [79]. It encompasses two conserved regions that form a pleated β-sheet sandwich, separated by a variable-length hydrophilic domain [80,81]. These structural features closely resemble those observed in the human and mouse NudC homologs [50], suggesting the possibility of common functions with sHSPs. In plants, ACD proteins serve diverse roles, from responding to abiotic stresses and hormones to regulating transcription, virus movement, and DNA demethylation [82]. The coordinated expression of different ACD proteins can collectively function as a chaperone network, safeguarding cellular machinery under a variety of stress conditions.

The Arabidopsis protein BOB1 demonstrates characteristics that align with typical sHSPs. It is induced under heat stress, possesses a NudC domain that shares structural homology with ACD-containing sHSPs, and exhibits in vitro chaperone activity, effectively preventing the aggregation of model protein substrates [39,80,83]. Under normal conditions, BOB1 is primarily localized in the plant cell cytoplasm, but during heat stress, it translocates to heat shock granules in association with HSP17.6 [39,84,85]. Previous studies have documented the presence of sHSPs, Hsp70s, and heat shock factors within these granules, which are hypothesized to serve as locations for interactions between misfolded proteins and chaperones [39,83,86,87,88]. It is interesting to note that heat shock granules can form in response to protein misfolding even in the absence of elevated temperatures [89]. The partial loss-of-function mutant *bob1-3* displays reduced thermotolerance, which can be rescued by introducing a functional *BOB1* transgene, underscoring the pivotal role of this NudC family member in enhancing plant thermotolerance [39].

BOB1, with its sHSP-like characteristics and crucial role in heat stress response, is not only involved in thermotolerance but also contributes to proteostasis. In conjunction with the 26S proteasome (26SP), it forms part of a genetic network linking proteostasis to the AS1-AS2 developmental pathway [67]. The interactions within this network are believed to rely on BOB1 chaperone activities. Importantly, this network plays a vital role in repressing *KNOX* gene expression, which is critical for normal plant development. These studies highlight the intricate link between heat stress response, proteostasis, and developmental processes in plants.

The second *NudC* gene in Arabidopsis, *NMig1*, also shares structural homology with ACD-containing sHSPs, and exhibits significant upregulation in response to various abiotic stressors, including heat shock [40]. Constitutive overexpression of *NMig1* results in enhanced root growth and lateral root development, even under adverse abiotic stress conditions. *NMig1*-overexpressing plants display reduced susceptibility to the inhibitory effects of abiotic stress on root morphology. Delving into the molecular mechanisms underlying these effects reveals elevated expression levels of genes encoding antioxidant enzymes and other genes closely associated with stress responses. The increased antioxidant activity, coupled with lower levels of reactive oxygen species (ROS) and reduced lipid peroxidation, underscores the critical role of *NMig1* in mitigating the impact of abiotic stress. Furthermore, *NMig1* overexpression coincides with an upregulation of heat shock proteins and genes related to abiotic stress responses, indicating its multifaceted role in enhancing plant stress resilience.

The involvement of tomato NudC homologs in the context of tomato immune responses is notably intriguing, given the broader context of the role of *NudC* genes in enhancing plant stress resilience as discussed earlier. In a study conducted by Liu et al. [68], a compelling connection between tomato orthologues of NudC domain proteins and SlSAP3, a member of the stress-associated protein family, came to light. SlSAP3 serves as a positive regulator of tomato immunity against *Pseudomonas syringae* pv. *tomato* (*Pst*) DC3000. The three identified tomato SlBOB proteins, namely SlBOB1, SlBOB2, and SlBOB3, share a common NudC domain at their C termini, with variations in regions outside the NudC domain [68]. Silencing *SlBOB1* or the simultaneous silencing of all three *SlBOB* genes, which act as negative regulators of immunity, leads to enhanced resistance against *Pst* DC3000. Hence, it appears that plant BOB proteins play diverse roles in responding to biotic and abiotic stress.

These findings further emphasize the vital role played by *NudC* genes in modulating plant stress resilience, and provide valuable insights into their potential as targets for the development of stress-tolerant crops.

## 4. Future Avenues of Research on NudC Proteins in Plants

**Figure 5:**
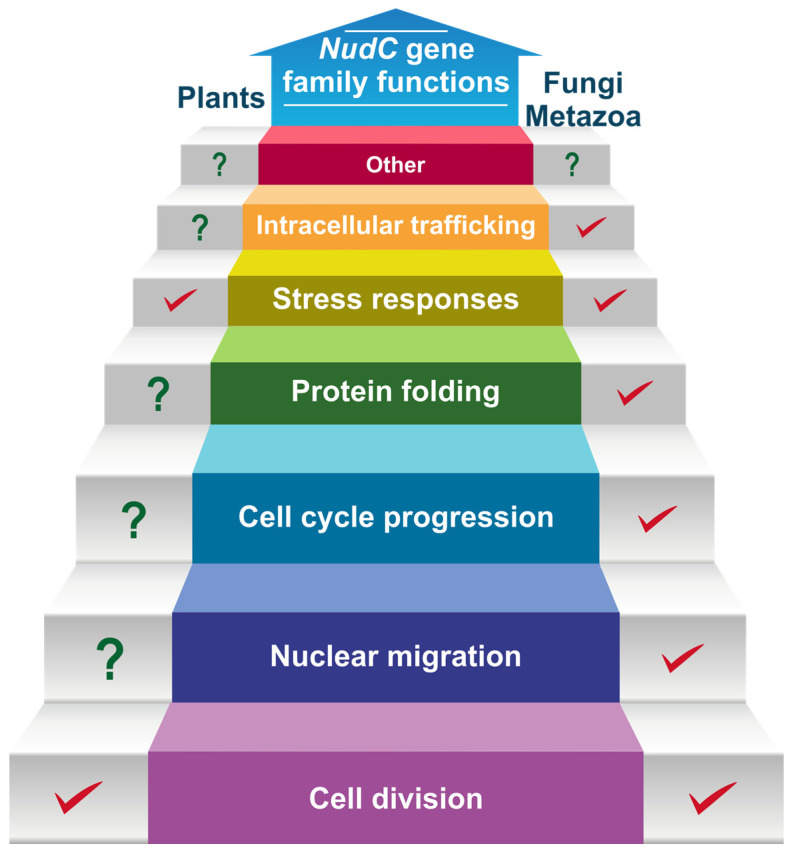
Hypothetical model representing the known and putative functions of *NudC* genes in different eukaryotes. Red check marks denote established functions supported by experimental evidences. Green question marks indicate predicted functions that still need to be explored.

Although some progress has been achieved in deciphering the molecular mechanisms underpinning the impact of NudC proteins on plants, many key questions remain on the horizon for forthcoming investigations (summarized in Figure 5). Research efforts are poised to examine the intricate mechanisms through which NudC members contribute to various aspects of plant morphology, growth, development, and stress responses. This exploration may encompass their involvement in diverse metabolic pathways, signal transduction processes, or interactions with other cellular constituents, extending beyond their currently recognized roles. Our research findings thus far have shed light on the vital role of NudC family members in the development and branching of the primary root in Arabidopsis, as well as in enhancing plant resistance against adverse abiotic stress factors [40,69]. Such studies rely on the development of an exhaustive genetic toolbox comprising transcriptional and translational reporters for *NMig1* and *BOB1*, as well as Arabidopsis lines with gene overexpression or downregulation in combination with tissue- and developmental-stage-specific promoters. Investigating the NudC interactions with specific protein partners which play an essential role in plant growth, development, and stress responses is of utmost importance. The dissection of the NudC protein interactome requires the generation of NudC gene versions with affinity purification tags, as well as fluorescent protein-tagged versions for further validation of the putative protein–protein interactions. Besides the interactomic studies, other systems biology approaches should also provide an added value to our knowledge of the role of plant NudC proteins. As outlined above, there is already some experimental evidence for the direct involvement of BOB1 in auxin-mediated processes. Hence, hormone profiling in lines with altered *NudC* expression will associate the observed growth phenotypes with the changes in the composition and quantity of endogenous phytohormones and their precursors, conjugates, and degradation products. Such findings, particularly their connection to temperature fluctuations and hormonal responses, offer the potential to unveil novel and essential aspects of plant functioning.

An additional avenue of research may involve in-depth structural analyses of NudC domains in plant species to elucidate differences in structural features of plant NudC domains compared to other organisms, and how these distinctions correlate with plant-specific functions. A possible approach in this respect could be the design of chimeric NudC versions with swapped protein domains from other eukaryotic organisms. The expression of such gene constructs in Arabidopsis mutants with impaired NudC gene expression would be instrumental in addressing the capability for functional complementation based on conserved protein signatures. This notwithstanding, expression of plant *NudC* genes in heterologous systems, such as yeast and mammalian cells, would shed light on the extent of functional homology when it comes to cell cycle progression.

The distinctive chaperone activities of NudC proteins in plants warrant comprehensive exploration. It appears that the members of this protein family might orchestrate the functioning of many HSP proteins through differential interactions depending on the environmental context. Further experiments with truncated protein versions and with the aforementioned chimeric constructs would elucidate the significance of specific protein motifs for the composition of the NudC protein interactome within the heat shock granules. A comprehensive understanding of the plant-specific chaperone network involving NudC proteins will further illuminate the molecular mechanisms of plant stress resilience. In addition to this, the advances in next-generation sequencing technologies have recently enabled the dissection of heat stress regulation modules [90]. Such thorough studies of the transcriptional signatures would shed light on the *NudC* gene coexpression networks in response to unfavorable conditions.

In summary, the ongoing studies on NudC proteins in plants hold the promise of revealing a wealth of knowledge that can significantly advance our comprehensive understanding of regulatory mechanisms at the whole-plant level. The obtained information could ultimately fuel novel strategies for enhancing crop productivity and strengthening plant resilience in response to environmental challenges. Notably, the structural and functional conservation of NudC proteins across species hints at their potential applications in medical research. Investigating NudC roles in cell division, microtubule regulation, and related pathways may lead to insights into human diseases and therapeutic opportunities. Realizing the full potential of NudC proteins necessitates further research and interdisciplinary collaboration. These endeavors hold the key to unlocking the profound benefits that NudC proteins can offer to plant biology and medical science.

## Supplementary Materials

The following supporting information can be downloaded at: https://www.mdpi.com/article/10.3390/plants13010119/s1, Table S1: Retrieved *AtBOB1* homologs across eukaryotes; Figure S1: Conserved domains on selected NudC proteins.
