# Supplementary material for: Small Sized Yet Powerful: Nuclear Distribution C Proteins in Plants
Source: Plants (Basel). 2023 Dec 31;13(1):119. doi: 10.3390/plants13010119 (PMC10780334; doi:10.3390/plants13010119)
Supplement: Supplementary file 1 [file plants-13-00119-s001.zip › plants-2746456-supplementary/Supplementary materials/Figure S1_Vassileva et al.pdf]

Conserved domains on selected NudC proteins

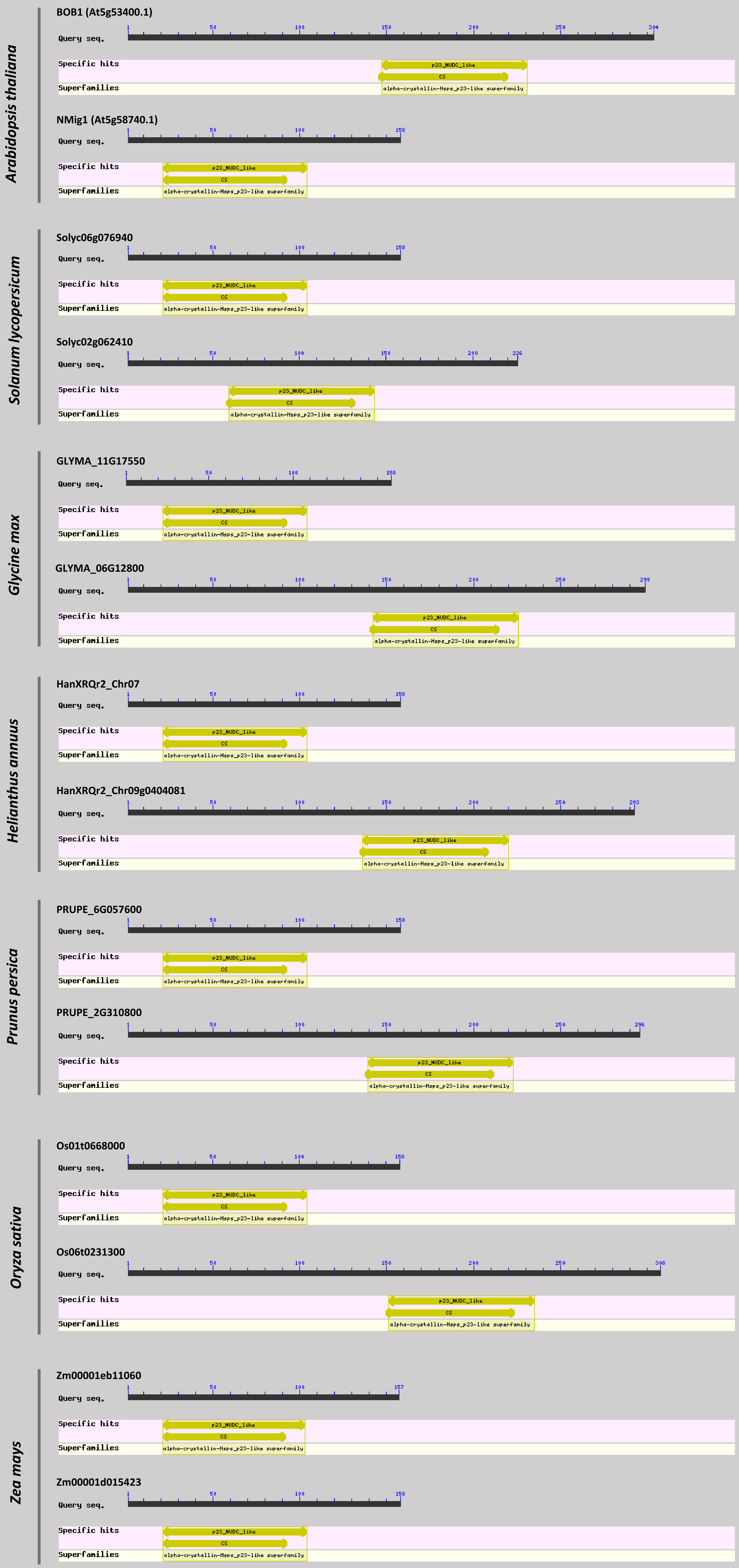

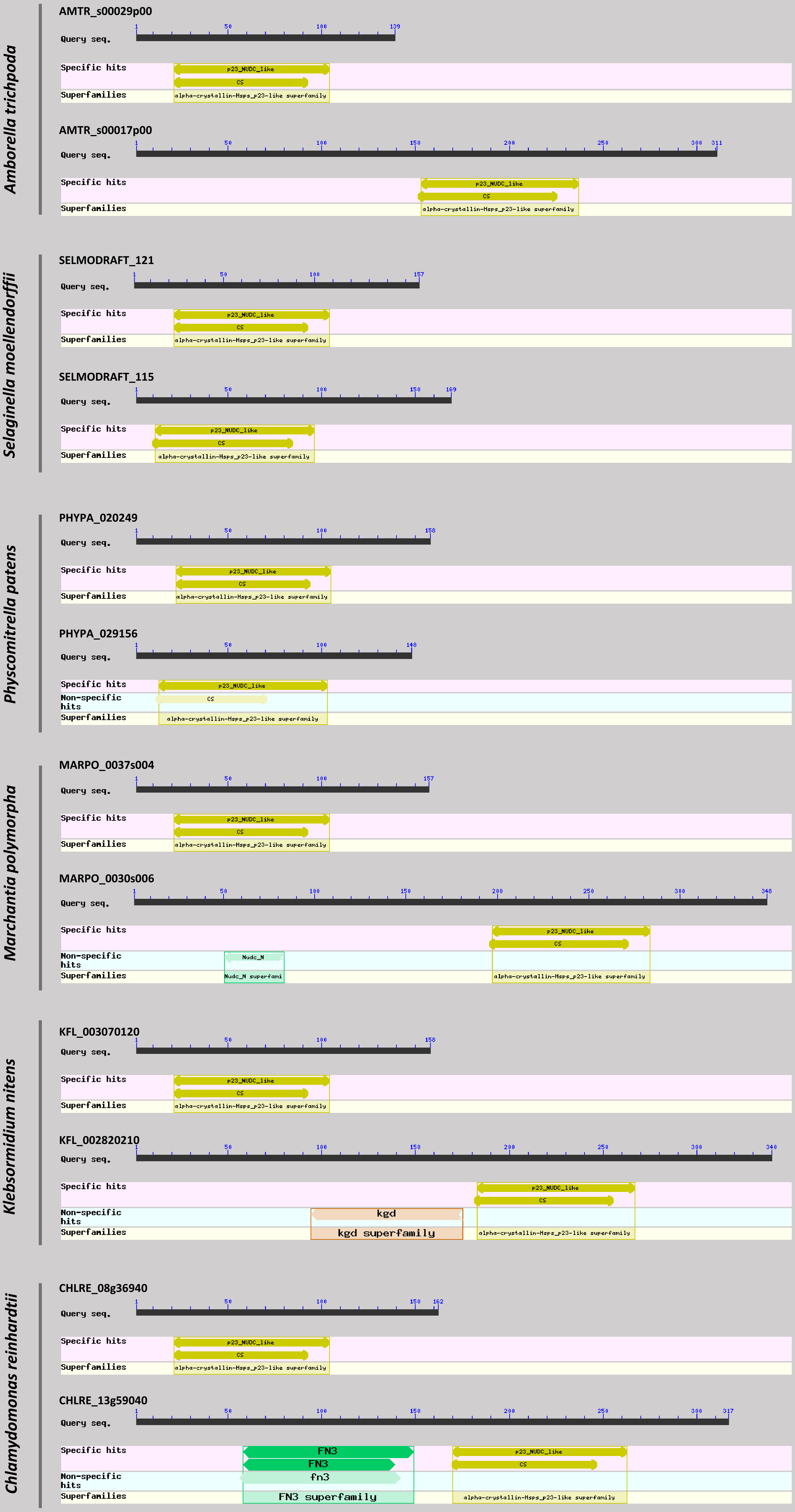
Amborella trichpoda
AMTR_s00029p00
Query seq.
Specific hits
Superfamilies
p23_NUDC_like
CS
alpha-crystallin-Hsps_p23-like superfamily
AMTR_s00017p00
Selaginella moellendorffii
SELMODRAFT_121
SELMODRAFT_115
Physcomitrella patens
PHYPA_020249
PHYPA_029156
Non-specific hits
Marchantia polymorpha
MARPO_0037s004
MARPO_0030s006
Nudc_N
Nudc_N superfami
Klebsormidium nitens
KFL_003070120
KFL_002820210
kgd
kgd superfamily
Chlamydomonas reinhardtii
CHLRE_08g36940
CHLRE_13g59040
FN3
fn3
FN3 superfamily
