# Supplementary material for: Small Sized Yet Powerful: Nuclear Distribution C Proteins in Plants
Source: Plants (Basel). 2023 Dec 31;13(1):119. doi: 10.3390/plants13010119 (PMC10780334; doi:10.3390/plants13010119)
Supplement: Supplementary file 1 [file plants-13-00119-s001.zip › plants-2746456-supplementary/Supplementary materials/Table S1_Vassileva et al.pdf]

Table S1. Retrieved *AtBOB1* homologs across eukaryotes

AtBOB1 homologs in plants and algae

Organism	Locus ID	Gene name	Uniprot ID	Number of exons	Protein length (aa)	Protein domains & positions	Maximum organ expression	Intracellular localization (found/predicted)	Functions (found/predicted)	References
Arabidopsis thaliana	*At4g27890*	*BOBBER 2 (BOB2)*	A0A5S9XWX5, Q9STN7	3	293	CS (131-220); p23_NUDC_like (136-220)	root, stem, flower, silique, seed	cytoplasm	unfolded protein binding, protein folding, response to stress	Jurkuta et al. (2009); Perez et al. (2009)
Arabidopsis thaliana	*At5g53400*	*BOBBER1 (BOB1)*	Q9LV09	6	304	CS (142-231); p23_NUDC_like (147-231)	root, shoot, flower, silique, seed	cytoplasm, cytoplasmic granules	protein folding, response to heat, pollen development, response to high light intensity, embryonic pattern specification, leaf morphogenesis, inflorescence meristem growth, developmental process, response to endoplasmic reticulum stress, response to hydrogen peroxide, stamen morphogenesis, specification of floral organ number	Jurkuta et al. (2009); Perez et al. (2009); Kaplinsky (2009)
Arabidopsis thaliana	*At5g58740*	*NMig1*	Q8VXX3, A0A178UER9	5	158	CS (16-104); p23_NUDC_like (21-104)	root, flower, silique, seed	cytoplasm, nucleus	unfolded protein binding, protein folding, response to stress, root development	Velinov et al. (2020); Velinov et al. (2021)
Brassica napus	*GSBRNA2T00004948001*	*BnaA10g29540D*	A0A078IM25	5	158	CS (16-104); p23_NUDC_like (21-104)		cytoplasm	unfolded protein binding, protein folding, response to stress	
Brassica napus	*GSBRNA2T00044314001*	*BnaA03g48890D*	A0A078GZ49	2	287	CS (126-215); p23_NUDC_like (131-215)		cytoplasm	unfolded protein binding, protein folding, response to stress	
Brassica napus	*GSBRNA2T00044313001*	*BnaA03g48880D*	A0A078GXE8	2	308	CS (148-238); p23_NUDC_like (153-235)		cytoplasm	unfolded protein binding, protein folding, response to stress	
Brassica napus	*GSBRNA2T00094192001*	*BnaC03g34480D*	A0A078IJ54	2	209	CS (47-136); p23_NUDC_like (52-133)		cytoplasm	unfolded protein binding, protein folding, response to stress	
Brassica napus	*GSBRNA2T00006266001*	*BnaC01g20510D*	A0A078G219	3	226	CS (64-153); p23_NUDC_like (69-153)		cytoplasm	unfolded protein binding, protein folding, response to stress	
Brassica rapa subsp. pekinensis	*Bra002621*	*Bra002621*	A0A397XSX1	5	158	CS (16-104); p23_NUDC_like (21-104)		cytoplasm	response to stress	
Brassica rapa subsp. pekinensis	*Bra040837*	*Bra040837*	M4FIA8	2	290	CS (129-218); p23_NUDC_like (134-218)			unfolded protein binding, protein folding, response to stress	
Brassica rapa subsp. pekinensis	*Bra040836*	*Bra040836*	A0A397L8K8	1	305	CS (145-235); p23_NUDC_like (150-232)			response to stress	

Brassica rapa subsp. pekinensis	*Bra026300*	*Bra026300*	M4EBZ1	2	289	CS (127-216); p23_NUDC_like (132-216)		cytoplasm	unfolded protein binding, protein folding, response to stress	
Brassica rapa subsp. pekinensis	*Bra003059*	*Bra003059*	M4CFS7	6	279	CS (117-206); p23_NUDC_like (122-206)		cytoplasm	unfolded protein binding, protein folding, response to stress	
Capsicum annuum	*T459_18329*	*T459_18329*	A0A1U8HBB2	5	158	CS (16-104); p23_NUDC_like (21-104)		cytoplasm, nucleus	unfolded protein binding, protein folding, response to stress	
Capsicum annuum	*T459_07419*	*T459_07419*	A0A2G2ZTM5	1	153	CS (25-120); p23_NUDC_like (30-120)		cytoplasm	unfolded protein binding, protein folding, response to stress	
Capsicum annuum	*T459_07420*	*T459_07420*	A0A2G2ZTK9	2	245	CS (60-149); p23_NUDC_like (65-149)		cytoplasm	unfolded protein binding, protein folding, response to stress	
Capsicum annuum	*T459_27622*	*T459_27622*	A0A2G2YEH3	1	117	NudC (6-110); HSP20-like chaperone (4-73)		cytoplasm	unfolded protein binding, protein folding	
Capsicum annuum	*T459_07418*	*T459_07418*	A0A2G2ZTL8	2	254	CS (82-171); p23_NUDC_like (87-171)		cytoplasm	unfolded protein binding, protein folding, response to stress	
Capsicum annuum	*T459_16487*	*T459_16487*	A0A2G2Z984	6	286	CS (124-213); p23_NUDC_like (129-213)		cytoplasm	unfolded protein binding, protein folding, response to stress	
Capsicum annuum	*T459_09591*	*T459_09591*	A0A1U8FZY1	6	307	CS (145-234); p23_NUDC_like (150-234)		cytoplasm	unfolded protein binding, protein folding, response to stress	
Lactuca sativa	*LSAT_2X122940*	*LSAT_2X122940*	A0A2J6M261	5	158	CS (16-104); p23_NUDC_like (21-104)		cytoplasm	unfolded protein binding, protein folding, response to stress	
Lactuca sativa	*LSAT_9X56360*	*LSAT_9X56360*	A0A2J6KDI6	6	306	CS (144-233); p23_NUDC_like (149-233)		cytoplasm	unfolded protein binding, protein folding, response to stress	
Solanum lycopersicum	*Solyc06g076940.3*	*SlBOBBER1 (SlBOB1)*	A0A3Q7HXJ3, K4CA52	5	158	CS (16-104); p23_NUDC_like (21-104)	root, fruit, seed	cytoplasm	unfolded protein binding, protein folding, developmental process, immune response	Liu et al. (2019)
Solanum lycopersicum	*Solyc09g092210.3*	*Solyc09g092210.3*	A0A3Q7I8N0	3	205	CS (58-147); p23_NUDC_like (63-147)	flower, fruit	cytoplasm	unfolded protein binding, protein folding, response to stress	
Solanum lycopersicum	*Solyc02g062410.2*	*SlBOBBER2 (SlBOB2)*	A0A3Q7FJG8	2	226	CS (54-143); NudC (10-204); p23_NUDC_like (59-143)	flower, fruit	cytoplasm	unfolded protein binding, protein folding, response to stress	Liu et al. (2019)
Solanum lycopersicum	*Solyc09g092200.2*	*Solyc09g092200.2*	A0A3Q7I8G7	4	219	CS (39-135); p23_NUDC_like (44-135)	flower, fruit	cytoplasm	unfolded protein binding, protein folding, response to stress	
Solanum lycopersicum	*Solyc06g051950.3*	*SlBOBBER3 (SlBOB3)*	A0A3Q7HNZ6	9	347	CS (191-280); p23_NUDC_like (196-280)	flower, fruit, seed	cytoplasm	response to stress, immune response	Liu et al. (2019)

Solanum lycopersicum	*Solyc03g083390.3*	*101255475*	K4BIB5, A0A3Q7FN48	6	302	CS (140-229); p23_NUDC_like (145-229)	stem, flower, fruit, seed	cytoplasm	unfolded protein binding, protein folding, response to stress	
Solanum tuberosum	*PGSC0003DMG400030355*	*102588480*	M1CZA6	5	158	CS (16-104); p23_NUDC_like (21-104)		cytoplasm, nucleus	unfolded protein binding, protein folding, response to stress	
Solanum tuberosum	*PGSC0003DMG400026423*	*PGSC0003DMG400026423*	M1CI53	2	247	CS (62-151); p23_NUDC_like (67-151)		cytoplasm	unfolded protein binding, protein folding, response to stress	
Solanum tuberosum	*PGSC0003DMG400046575*	*PGSC0003DMG400046575*	M1DZP7	2	211	CS (39-128); p23_NUDC_like (44-128)		cytoplasm	unfolded protein binding, protein folding, response to stress	
Solanum tuberosum	*PGSC0003DMG400009123*	*102577908*	Q38HV0	6	308	CS (146-235); p23_NUDC_like (151-235)		cytoplasm	unfolded protein binding, protein folding, response to stress	
Nicotiana tabacum	*LOC107815638*	*LOC107815638*	A0A1S4C715	5	158	CS (16-104); p23_NUDC_like (21-104)		cytoplasm	unfolded protein binding, protein folding, response to stress	
Nicotiana tabacum	*LOC107796635*	*LOC107796635*	A0A1S4AEN1	5	158	CS (16-104)		cytoplasm	unfolded protein binding, protein folding, response to stress	
Nicotiana tabacum	*LOC107769666*	*LOC107769666*	A0A1S3XWY9	1	170	CS (49-138)		cytoplasm	unfolded protein binding, protein folding, response to stress	
Nicotiana tabacum	*LOC107769667*	*LOC107769667*	A0A1S3XWY7	2	251	CS (65-154); p23_NUDC_like (54-138)		cytoplasm	unfolded protein binding, protein folding, response to stress	
Nicotiana tabacum	*LOC107785569*	*LOC107785569*	A0A1S3ZD95	1	230	CS (57-146); p23_NUDC_like (62-146)		cytoplasm	unfolded protein binding, protein folding, response to stress	
Nicotiana tabacum	*LOC107779500*	*LOC107779500*	A0A1S3YTB1	1	213	CS (46-135)		cytoplasm	unfolded protein binding, protein folding, response to stress	
Nicotiana tabacum	*LOC107791897*	*LOC107791897*	A0A1S3ZYN1	2	166	CS (46-135)		cytoplasm	unfolded protein binding, protein folding, response to stress	
Nicotiana tabacum	*LOC107790710*	*LOC107790710*	A0A1S3ZUT2	2	240	CS (67-156)		cytoplasm	unfolded protein binding, protein folding, response to stress	
Nicotiana tabacum	*LOC107816713*	*LOC107816713*	A0A1S4CA10	6	289	CS (127-216); p23_NUDC_like (132-216)		cytoplasm	unfolded protein binding, protein folding, response to stress	
Nicotiana tabacum	*LOC107775992*	*LOC107775992*	A0A1S3YGN1	6	299	CS (137-226)		cytoplasm	unfolded protein binding, protein folding, response to stress	
Nicotiana tabacum	*LOC107798015*	*LOC107798015*	A0A1S4AIB2	6	301	CS (139-228); p23_NUDC_like (144-228)		cytoplasm	unfolded protein binding, protein folding, response to stress	
Cucumis sativus	*Csa1G530660*	*Csa1G530660*	A0A0A0LV57	5	156	CS (16-104); p23_NUDC_like (21-104)		cytoplasm, nucleus	unfolded protein binding, protein folding, response to stress	
Cucumis sativus	*Csa5G139440*	*Csa5G139440*	A0A0A0KQ62	6	315	CS (153-242); p23_NUDC_like (158-242)		cytoplasm	unfolded protein binding, protein folding, response to stress	
Medicago truncatula	*MTR_3g463620*	*MTR_3g463620*	A0A072UXE1, I3SFI1	4	180	CS (44-132); p23_NUDC_like (49-132)	root	cytoplasm, nucleus	response to stress	

Medicago truncatula	*MTR_1g008195*	*25481856*	A0A072VDR8	6	290	CS (128-217); p23_NUDC_like (133-217)	seed	cytoplasm	unfolded protein binding, protein folding, response to stress	
Medicago truncatula	*MTR_1g008170*	*11431318*	G7I4A6	6	289	CS (128-217); p23_NUDC_like (133-217)	seed	cytoplasm	unfolded protein binding, protein folding, response to stress	
Medicago truncatula	*MTR_1g008200*	*11429600*	G7I4A8	6	295	CS (133-222); p23_NUDC_like (138-222)	nodule	cytoplasm	unfolded protein binding, protein folding, response to stress	
Glycine max	*GLYMA_11G175500*	*GLYMA_11G175500*	C6SYT5, I1LLK0	5	158	CS (16-104); p23_NUDC_like (21-104)	leaf, seed	cytoplasm	unfolded protein binding, protein folding, response to stress	
Glycine max	*GLYMA_18G063100*	*100306544*	C6SZU0	5	158	CS (16-104); p23_NUDC_like (21-104)	nodule, seed, embryo	cytoplasm	unfolded protein binding, protein folding, response to stress	
Glycine max	*GLYMA_06G128000*	*100775670*	I1KAR6	6	299	CS (137-226); p23_NUDC_like (142-226)	nodule, embryo	cytoplasm	unfolded protein binding, protein folding, response to stress	
Glycine max	*GLYMA_04G236300*	*100813799*	C6TKE3	6	301	CS (139-228); p23_NUDC_like (144-228)	nodule, embryo	cytoplasm	unfolded protein binding, protein folding, response to stress	
Eucalyptus grandis	*EUGRSUZ_B02255*	*EUGRSUZ_B02255*	A0A059D4G2	5	221	CS (79-167); p23_NUDC_like (84-167)		cytoplasm, nucleus	unfolded protein binding, protein folding, response to stress	
Eucalyptus grandis	*EUGRSUZ_A01460*	*EUGRSUZ_A01460*	A0A059DEY7	7	407	CS (245-334); p23_NUDC_like (250-334)		cytoplasm, membrane	unfolded protein binding, protein folding, response to stress	
Juglans regia	*LOC109002082*	*LOC109002082*	A0A6P9EDV6	6	180	CS (24-123); p23_NUDC_like (29-123)		cytoplasm	unfolded protein binding, protein folding, response to stress	
Juglans regia	*LOC109015374*	*LOC109015374*	A0A6P9EFR4	5	158	CS (16-104); p23_NUDC_like (21-104)		cytoplasm	unfolded protein binding, protein folding, response to stress	
Juglans regia	*LOC108980108*	*LOC108980108*	A0A2I4DH47	1	280	CS (118-207); p23_NUDC_like (123-207)		cytoplasm, nucleus	unfolded protein binding, protein folding, response to stress	
Juglans regia	*LOC108982868*	*LOC108982868*	A0A2I4DRW5	6	296	CS (134-223); p23_NUDC_like (139-223)		cytoplasm	unfolded protein binding, protein folding, response to stress	
Juglans regia	*LOC109013205*	*LOC109013205*	A0A2I4H3N7	6	296	CS (134-223); p23_NUDC_like (139-223)		cytoplasm	unfolded protein binding, protein folding, response to stress	
Theobroma cacao	*TCM_041625*	*TCM_041625*	A0A061GV77	5	158	CS (16-104); p23_NUDC_like (21-104)		cytoplasm	unfolded protein binding, protein folding, response to stress	
Theobroma cacao	*TCM_021712*	*TCM_021712*	A0A061EYB6	1	174	CS (12-101); p23_NUDC_like (18-101)		cytoplasm	unfolded protein binding, protein folding, response to stress	
Theobroma cacao	*TCM_015401*	*TCM_015401*	A0A061G2U6	6	295	CS (133-222); p23_NUDC_like (138-222)		cytoplasm	unfolded protein binding, protein folding, response to stress	
Manihot esculenta	*MANES_02G119500*	*MANES_02G119500*	A0A2C9WD44	5	158	CS (16-104); p23_NUDC_like (21-104)		cytoplasm	unfolded protein binding, protein folding, response to stress	
Manihot esculenta	*MANES_01G161700*	*MANES_01G161700*	A0A2C9WLD1	5	158	CS (16-104); p23_NUDC_like (21-104)		cytoplasm	unfolded protein binding, protein folding, response to stress	

Manihot esculenta	*MANES_14G006200*	*MANES_14G006200*	A0A2C9UIQ0	6	320	CS (158-247); p23_NUDC_like (163-247)		cytoplasm	unfolded protein binding, protein folding, response to stress	
Manihot esculenta	*MANES_06G160400*	*MANES_06G160400*	A0A2C9VRD1	4	286	CS (223-286); p23_NUDC_like (228-286)		cytoplasm, membrane	unfolded protein binding, protein folding, response to stress	
Manihot esculenta	*MANES_06G160000*	*MANES_06G160000*	A0A2C9VST6	3	252	CS (178-252); p23_NUDC_like (183-244)			response to stress	
Prunus persica	*PRUPE_6G057600*	*PRUPE_6G057600*	M5W0J7	5	158	CS (16-104); p23_NUDC_like (21-104)		cytoplasm, nucleus	unfolded protein binding, protein folding, response to stress	
Prunus persica	*PRUPE_2G310800*	*PRUPE_2G310800*	M5XEL2	6	296	CS (16-104); p23_NUDC_like (21-104)		cytoplasm, endoplasmic reticulum, membrane	calcium ion binding, unfolded protein binding, unfolded protein binding, endoplasmic reticulum calcium ion homeostasis,protein folding, response to stress	
Erythranthe guttata	*MIMGU_mgv1a015421mg*	*MIMGU_mgv1a015421mg*	A0A022RC76	6	158	CS (16-104); p23_NUDC_like (21-104)		cytoplasm	unfolded protein binding, protein folding, response to stress	
Erythranthe guttata	*MIMGU_mgv1a010735mg*	*MIMGU_mgv1a010735mg*	A0A022PVU0	6	303	CS (141-230); p23_NUDC_like (146-230)		cytoplasm	unfolded protein binding, protein folding, response to stress	
Erythranthe guttata	*MIMGU_mgv1a011251mg*	*MIMGU_mgv1a011251mg*	A0A022QB71	6	288	CS (126-215); p23_NUDC_like (131-215)		cytoplasm	unfolded protein binding, protein folding, response to stress	
Helianthus annuus	*HannXRQ_Chr07g0202931*	*HannXRQ_Chr07g0202931*	A0A251UEM1	5	158	CS (16-104); p23_NUDC_like (21-104)		cytoplasm	unfolded protein binding, protein folding, response to stress	
Helianthus annuus	*HannXRQ_Chr08g0221811*	*HannXRQ_Chr08g0221811*	A0A251U4P2	5	158	CS (16-104); p23_NUDC_like (21-104)		cytoplasm	unfolded protein binding, protein folding, response to stress	
Helianthus annuus	*HannXRQ_Chr09g0267241*	*BOB1*	A0A251TY55	6	293	CS (131-220)		cytoplasm	unfolded protein binding, protein folding, response to stress	
Populus trichocarpa	*POPTR_003G100900v3*	*POPTR_003G100900*	B9GZU9	1	272	CS (103-192); p23_NUDC_like (109-192)		cytoplasm	unfolded protein binding, protein folding, response to stress	
Populus trichocarpa	*POPTR_001G132500v3*	*POPTR_001G132500*	A0A2K2BWX2	1	261	CS (104-193)			response to stress	
Populus trichocarpa	*POPTR_015G013900v3*	*POPTR_015G013900*	A0A2K1XGH3	6	300	CS (138-227)		cytoplasm	unfolded protein binding, protein folding, response to stress	
Populus trichocarpa	*POPTR_012G014100v3*	*POPTR_012G014100*	A0A2K1Y782	6	289	CS (127-216)		cytoplasm	unfolded protein binding, protein folding, response to stress	
Ricinus communis	*RCOM_0612440*	*RCOM_0612440*	B9SI21	6	158	CS (16-104); p23_NUDC_like (21-104)		cytoplasm	unfolded protein binding, protein folding, response to stress	
Ricinus communis	*RCOM_0169970*	*RCOM_0169970*	B9T3X1	3	87			cytoplasm	unfolded protein binding, protein folding	
Ricinus communis	*RCOM_0597500*	*RCOM_0597500*	B9SJ06	2	209	CS (47-136); p23_NUDC_like (53-135)		cytoplasm	unfolded protein binding, protein folding, response to stress	
Ricinus communis	*RCOM_1579230*	*RCOM_1579230*	B9RIH7	6	307	CS (151-240); p23_NUDC_like (156-240)		cytoplasm	unfolded protein binding, protein folding, response to stress	
Gossypium hirsutum	*LOC107936543*	*LOC107936543*	A0A1U8MGZ5	5	158	CS (16-104); p23_NUDC_like (21-104)		cytoplasm	unfolded protein binding, protein folding, response to stress	

Gossypium hirsutum	*LOC107891963*	*LOC107891963*	A0A1U8I2D0	5	158	CS (16-104); p23_NUDC_like (21-104)		cytoplasm	unfolded protein binding, protein folding, response to stress	
Gossypium hirsutum	*LOC107946804*	*LOC107946804*	A0A1U8NCM9	1	269	CS (107-196); p23_NUDC_like (111-194)		cytoplasm	unfolded protein binding, protein folding, response to stress	
Gossypium hirsutum	*LOC107947245*	*LOC107947245*	A0A1U8NE51	6	291	CS (129-218); p23_NUDC_like (134-218)			response to stress	
Gossypium hirsutum	*LOC107930277*	*LOC107930277*	A0A1U8LS30	6	289	CS (127-216); p23_NUDC_like (132-216)		cytoplasm	unfolded protein binding, protein folding, response to stress	
Gossypium hirsutum	*LOC107929622*	*LOC107929622*	A0A1U8LPU0	6	296	CS (134-223); p23_NUDC_like (139-223)			response to stress	
Gossypium hirsutum	*LOC107894867*	*LOC107894867*	A0A1U8IGS2	7	285	CS (123-212); p23_NUDC_like (128-212)		cytoplasm	unfolded protein binding, protein folding, response to stress	
Gossypium hirsutum	*LOC107944939*	*LOC107944939*	A0A1U8N5J0	1	270	CS (111-200); p23_NUDC_like (116-200)		cytoplasm	unfolded protein binding, protein folding, response to stress	
Gossypium hirsutum	*LOC107944267*	*LOC107944267*	A0A1U8N3B4	1	269	CS (111-200); p23_NUDC_like (116-200)		cytoplasm	unfolded protein binding, protein folding, response to stress	
Gossypium hirsutum	*LOC107890769*	*LOC107890769*	A0A1U8HXR4	6	289	CS (127-216); p23_NUDC_like (132-216)		cytoplasm	unfolded protein binding, protein folding, response to stress	
Vitis vinifera	*VIT_12s0142g00120*	*VIT_12s0142g00120*	D7TR32	6	158	CS (16-104); p23_NUDC_like (21-104)		cytoplasm	unfolded protein binding, protein folding, response to stress	
Vitis vinifera	*VIT_16s0098g00370*	*VIT_16s0098g00370*	E0CVH6	6	289	CS (127-216); p23_NUDC_like (132-216)		cytoplasm	unfolded protein binding, protein folding, response to stress	
Nelumbo nucifera	*LOC104601263*	*LOC104601263*	A0A1U8AKM9	5	130	CS (16-104); p23_NUDC_like (21-104)				
Nelumbo nucifera	*LOC104604013*	*LOC104604013*	A0A1U8AHB1	6	315	CS (153-242); p23_NUDC_like (158-242)		cytoplasm	unfolded protein binding, protein folding	
Musa acuminata subsp. malaccensis	*GSMUA_Achr6G27580_001 (Ma06_t29350.1)*	*103989249*	A0A804JLS5	5	158	CS (16-104)				
Musa acuminata subsp. malaccensis	*GSMUA_Achr2G07330_001 (Ma02_t09250.1)*	*103976403*	A0A804I0W5		282	CS (120-209)				
Musa acuminata subsp. malaccensis	*GSMUA_Achr11G13090_001 (Ma11_t11290.1)*	*103971323*	A0A804L6P6	6	306	CS (144-233); p23_NUDC_like (149-233)				
Brachypodium distachyon	*BRADI_5g07310v3*	*100828146*	I1IWY1	5	158	CS (16-104); p23_NUDC_like (21-104)		cytoplasm, nucleus	unfolded protein binding, protein folding	
Brachypodium distachyon	*BRADI_1g45080v3*	*100832845*	I1GZI6	6	322	CS (170-259); p23_NUDC_like (175-259)		cytoplasm	unfolded protein binding, protein folding	
Brachypodium distachyon	*BRADI_2g05060v3*	*100839208*	I1HCN3	6	183	CS (21-110); p23_NUDC_like (26-110)		cytoplasm	unfolded protein binding, protein folding	
Hordeum vulgare subsp. vulgare	*HORVU2Hr1G068470 (HORVU.MOREX.r3.2HG0161290)*		F2CY14	5	158	CS (16-104)		cytoplasm, nucleus	unfolded protein binding, protein folding	

Hordeum vulgare subsp. vulgare	*HORVU3Hr1G110760 (HORVU.MOREX.r3.3HG0323660)*		A0A8I6Y2S2	2	217	CS (54-143)				
Hordeum vulgare subsp. vulgare	*HORVU3Hr1G110720 (HORVU.MOREX.r3.3HG0323630)*		A0A8I6XCL8	1	214	CS (54-143)				
Hordeum vulgare subsp. vulgare	*HORVU7Hr1G038520 (HORVU.MOREX.r3.7HG0666750)*		F2E732	6	307	CS (145-234)				
Triticum aestivum	*TraesCS2A02G279900*		A0A3B6AZB0	5	158	CS (16-104); p23_NUDC_like (21-104)	root, endosperm, shoot axis, stigma and ovary	cytoplasm	unfolded protein binding, protein folding	
Triticum aestivum	*TraesCS2B02G296900*		A0A3B6C6B1	6	158	CS (16-104); p23_NUDC_like (21-104)	root, leaf, embryo	cytoplasm	unfolded protein binding, protein folding	
Triticum aestivum	*TraesCS2D02G278900*		A0A3B6DDH6	5	158	CS (16-104); p23_NUDC_like (21-104)	root	cytoplasm	unfolded protein binding, protein folding	
Triticum aestivum	*TraesCS3B02G566300*	*TRAES_3BF052700020CFD_c1*	W5D4R8	2	214	CS (51-140); p23_NUDC_like (56-140)		cytoplasm	unfolded protein binding, protein folding	
Triticum aestivum	*TraesCS3B02G566200*	*TRAES_3BF052700030CFD_c1*	W5D6E5	1	219	CS (56-145); p23_NUDC_like (61-145)		cytoplasm	unfolded protein binding, protein folding	
Triticum aestivum	*TraesCS3D02G509500*	*TraesCS3D02G509500*	A0A3B6H3F0	2	214	CS (51-140); p23_NUDC_like (56-140)		cytoplasm	unfolded protein binding, protein folding	
Triticum aestivum	*TraesCS3A02G503000*	*TraesCS3A02G503000*	A0A3B6ERA3	2	168	CS (48-137); p23_NUDC_like (53-137)		cytoplasm	unfolded protein binding, protein folding	
Triticum aestivum	*TraesCS3D02G509300*	*TraesCS3D02G509300*	A0A3B6H6D2	1	214	CS (51-140); p23_NUDC_like (56-140)		cytoplasm	unfolded protein binding, protein folding	
Triticum aestivum	*TraesCS3A02G502900*	*TraesCS3A02G502900*	A0A3B6EQH3	1	215	CS (52-141); p23_NUDC_like (57-141)		cytoplasm	unfolded protein binding, protein folding	
Triticum aestivum	*TraesCS7A02G189600*	*TraesCS7A02G189600*	A0A3B6RFV2	6	316	CS (154-243); p23_NUDC_like (159-243)	root, shoot, leaf, stigma and ovary, grain, endosperm, embryo			
Triticum aestivum	*TraesCS7B02G094500*	*TraesCS7B02G094500*	A0A3B6SDZ9	6	320	CS (158-247); p23_NUDC_like (163-247)	root, shoot, leaf, stigma and ovary, embryo	cytoplasm	unfolded protein binding, protein folding, response to stress	
Triticum aestivum	*TraesCS7D02G190700*	*TraesCS7D02G190700*	A0A3B6TMJ0	6	314	CS (152-241); p23_NUDC_like (157-241)	root, shoot, leaf, stigma and ovary, grain, endosperm, embryo			
Oryza sativa	*Os01g0668000*	*Os01g0668000*	B9EYI7	6	158	CS (16-104); p23_NUDC_like (21-104)		cytoplasm, nucleus	response to stress	
Oryza sativa	*Os02g0743800*	*Os02g0743800*	A0A0P0VPG4, Q6Z2U6	1	196					

Oryza sativa	*Os06g0231300*	*Os06g0231300*	A3B9X9	6	308	CS (146-235); p23_NUDC_like (151-235)				
Setaria italica	*SETIT_011893mg*	*SETIT_011893mg*	K3YCE7	5	301	CS (159-247); p23_NUDC_like (164-247)		cytoplasm	unfolded protein binding, protein folding	
Setaria italica	*SETIT_006940mg*	*101764727*	K3XYN4	6	285	CS (120-212); p23_NUDC_like (147-212)				
Sorghum bicolor	*SORBI_3001G301800*	*SORBI_3001G301800*	C5WSG2	5	158	CS (16-104); p23_NUDC_like (21-104)		cytoplasm	unfolded protein binding, protein folding	
Sorghum bicolor	*SORBI_3002G400200*	*SORBI_3002G400200*	C5X444	5	159	CS (16-105); p23_NUDC_like (21-105)		cytoplasm	unfolded protein binding, protein folding	
Sorghum bicolor	*SORBI_3001G520500*	*SORBI_3001G520500*	C5WZY5	6	181	CS (19-108); p23_NUDC_like (24-108)		cytoplasm	unfolded protein binding, protein folding	
Sorghum bicolor	*SORBI_3010G094500*	*SORBI_3010G094500*	A0A194YI58	7	264	CS (102-191); p23_NUDC_like (107-191)				
Zea mays	*Zm00001d015423*	*100283235*	B6T1R3	2	158	CS (16-104)	root, tassel, cob, internode tissue, seed	cytoplasm	unfolded protein binding, protein folding	
Zea mays	*Zm00001d007069*	*ZEAMMB73_Zm00001d007069*	A0A1D6F2Q5	7	162	p23_NUDC_like (1-73)	root, internode tissue, seed	cytoplasm	unfolded protein binding, protein folding, response to stress	
Zea mays	*Zm00001d044815*	*100282404*	B4G0G4	6	302	CS (140-229); p23_NUDC_like (145-229)	root, shoot, leaf, tassel, cob, seed, embryo		response to stress	
Zea mays	*Zm00001d037236*	*100273219*	B4FTP9	6	308	CS (146-235); p23_NUDC_like (151-235)	root, shoot, leaf, tassel, cob, seed, embryo	cytoplasm	unfolded protein binding, protein folding, response to stress	
Zostera marina	*ZOSMA_112G00430*	*ZOSMA_112G00430*	A0A0K9Q555	5	157	CS (16-104)		cytoplasm	unfolded protein binding, protein folding	
Zostera marina	*ZOSMA_191G00110*	*ZOSMA_191G00110*	A0A0K9PPK1	4	111			cytoplasm	unfolded protein binding, protein folding	
Zostera marina	*ZOSMA_325G00030*	*ZOSMA_325G00030*	A0A0K9PAX6	6	205	CS (77-166)		cytoplasm	unfolded protein binding, protein folding	
Zostera marina	*ZOSMA_21G00700*	*ZOSMA_21G00700*	A0A0K9PJT1	6	333	CS (171-260)		cytoplasm	unfolded protein binding, protein folding	
Amborella trichopoda	*AMTR_s00029p00243890*	*AMTR_s00029p00243890*	W1PIK0	4	139	CS (16-104); p23_NUDC_like (21-104)		cytoplasm, nucleus	unfolded protein binding, protein folding	
Amborella trichopoda	*AMTR_s00017p00231540*	*AMTR_s00017p00231540*	W1PM11	6	311	CS (148-237); p23_NUDC_like (21-104)		cytoplasm	unfolded protein binding, protein folding	
Selaginella moellendorffii	*SELMODRAFT_121870*	*SELMODRAFT_121870*	D8RGA0	5	157	CS (16-104); p23_NUDC_like (21-104)		cytoplasm	unfolded protein binding, protein folding	
Selaginella moellendorffii	*SELMODRAFT_115773*	*SELMODRAFT_115773*	D8SFG5	5	169	CS (6-96); p23_NUDC_like (11-96)		cytoplasm	unfolded protein binding, protein folding	
Selaginella moellendorffii	*SELMODRAFT_85440*	*SELMODRAFT_85440*	D8R5J0	5	169	CS (6-96); p23_NUDC_like (11-96)		cytoplasm	unfolded protein binding, protein folding	

Physcomitrella patens	*Pp3c16_190*	*PHYPA_020249*	A9SZZ2	6	158	CS (17-105); p23_NUDC_like (22-105)		cytoplasm, nucleus	unfolded protein binding, protein folding	
Physcomitrella patens	*Pp3c24_16270*	*PHYPA_029156*	A0A2K1IH15	3	148	CS (8-103); p23_NUDC_like (13-103)		cytoplasm	unfolded protein binding, protein folding	
Physcomitrella patens	*Pp3c11_14270*	*PHYPA_015017*	A0A2K1JUQ1	6	392	CS (230-319); p23_NUDC_like (235-319)		cytoplasm	unfolded protein binding, protein folding	
Physcomitrella patens	*Pp3c4_23090*	*PHYPA_006628*	A0A2K1KPQ5	6	472	CS (284-373); p23_NUDC_like (289-373)		cytoplasm	unfolded protein binding, protein folding	
Physcomitrella patens	*Pp3c2_28400*	*Pp3c2_28400*	A0A2K1L3C1	2	278	CS (194-236); p23_NUDC_like (195-240)				
Physcomitrella patens	*Pp3c1_7900*	*PHYPA_000356*	A0A2K1L7F1	7	362	CS (199-289); p23_NUDC_like (204-289)		cytoplasm	unfolded protein binding, protein folding	
Marchantia polymorpha	*MARPO_0037s0041*	*MARPO_0037s0041*	A0A2R6X445	5	157	CS (16-104); p23_NUDC_like (21-104)		cytoplasm, nucleus	unfolded protein binding, protein folding	
Marchantia polymorpha	*MARPO_0030s0062*	*MARPO_0030s0062*	A0A2R6X894	6	348	CS (186-275); p23_NUDC_like (191-275)		cytoplasm	unfolded protein binding, protein folding	
Klebsormidium nitens	*KFL_003070120*	*KFL_003070120*	A0A1Y1I6Z6	5	158	CS (16-104)		cytoplasm	unfolded protein binding, protein folding	
Klebsormidium nitens	*KFL_002820210*	*KFL_002820210*	A0A1Y1I5U3	6	340	CS (178-267)		cytoplasm	unfolded protein binding, protein folding	
Chlamydomonas reinhardtii	*CHLRE_08g369400v5*	*CHLRE_08g369400v5*	A8J474	5	162	CS (16-104); p23_NUDC_like (21-104)		cytoplasm	unfolded protein binding, protein folding	
Chlamydomonas reinhardtii	*CHLRE_13g590400v5*	*CHLRE_13g590400v5*	A8IW64	7	317	CS (165-263); p23_NUDC_like (170-263)		cytoplasm	unfolded protein binding, protein folding	
Chlamydomonas reinhardtii	*CHLRE_07g329600v5*	*CHLRE_07g329600v5*	A0A2K3DJS4	10	1102	CS (951-1033); p23_NUDC_like (951-1033)				
Chlamydomonas reinhardtii	*CHLRE_17g711650v5*	*CHLRE_17g711650v5*	A0A2K3CPM2	8	343	CS (182-270); p23_NUDC_like (187-270)		cytoplasm		

AtBOB1 homologs in other eukaryotic systems

Organism	Locus ID (NCBI)	Gene name	Uniprot ID	Number of exons	Protein length (aa)	Protein domain & position	Intracellular localization	Functions	References
Homo sapiens	*30535*	*NUDCD2*	Q8WVJ2	4	157	CS (14-104); p23_NUDC_like (12-104)	cytoplasm, cytosol, intercellular bridge, kinetochore, microtubule cytoskeleton, microtubule organizing center, mitotic spindle, spindle pole	unfolded protein binding, protein folding	Burkard et al. (2011); Scheuerle et al. (2023); Chen et al. (2020); Li et al. (2019)
Homo sapiens	*22208*	*NUDCD3*	Q8IVD9	6	361	CS (185-277); p23_NUDC_like (184-285)	cytoplasm, cytoplasm dynein complex	unfolded protein binding, cilium assembly, protein folding, protein localization to pericentriolar material	Rose et al. 2010; Cai et al. 2009; Zhou et al. 2006
Homo sapiens	*8045*	*NUDC*	Q9Y266	9	331	CS (167-258); p23_NUDC_like (172-258)	cytoplasm, cytosol, intercellular bridge, microtubule, midbody, mitotic spindle, nucleoplasm, spindle	cadherin binding, unfolded protein binding, cell division, mitotic metaphase chromosome alignment, mitotic spindle organization, nuclear migration, protein folding, response to peptide hormone	Miller et al. (1999); Matsumoto and Ledbetter (1999); Zhou et al. (2003); Aumais et al. (2003); Chen et al. (2015); Biebl et al. (2022); Islam et al. (2020); Bagci et al. (2020); Weiderhold et al. (2016); Zhou et al. (2016); Gladwyn-Ng et al. (2016); Gladwyn-Ng et al. (2015); De Souza et al. (2014); Chuang et al. (2013); Zhu et al. (2010); Pang et al. (2009); Tang et al. (2008); Lin et al. (2004)
Mus musculus	*MGI=1277103*	*Nudcd2*	Q9CQ48	4	157	CS (19-91); p23_NUDC_like (12-104)	cytoplasm, cytosol, intercellular bridge, kinetochore, microtubule cytoskeleton, microtubule organizing center, mitotic spindle, spindle pole	unfolded protein binding, protein folding	Skarnes et al. (2011)
Mus musculus	*MGI=2144158*	*Nudcd3*	Q8R1N4	6	363	CS (187-279); p23_NUDC_like (186-287)	cytoplasm, cytoplasmic dynein complex	cilium assembly, protein folding, protein localization to pericentriolar material	Yang et al. (2022)
Mus musculus	*MGI=106014*	*Nudc*	O35685	8	332	CS (168-259); p23_NUDC_like (173-259)	cytoplasm, cytosol, microtubule, midbody, mitotic spindle, nucleus	unfolded protein binding, cell division, mitotic metaphase chromosome alignment, mitotic spindle organization, nuclear migration, protein folding, response to peptide hormone	Aumais et al. (2001); Yamada et al. (2010)
Rattus norvegicus	*1307203*	*Nudcd2*	Q5M823	5	157	CS (14-104); p23_NUDC_like (12-104)	cytoplasm, cytosol, intercellular bridge, kinetochore, microtubule organizing center, mitotic spindle, spindle pole	unfolded protein binding, protein folding	
Rattus norvegicus	*6493297*	*LOC100911422*	Q63525	8	332	CS (168-259); p23_NUDC_like (173-259)	cytoplasm, cytosol, microtubule, midbody, mitotic spindle, nucleus	unfolded protein binding, cell division, mitotic metaphase chromosome alignment, mitotic spindle organization, nuclear migration, protein folding	Morris et al. (1997); Morris and Yu-Lee (1998)
Rattus norvegicus	*3215*	*Nudc*	A0A0G2K0V8	8	316	CS (152-243); p23_NUDC_like (157-243)	microtubule, midbody, spindle	cell cycle, cell division	Morris et al. (1997); Morris and Yu-Lee (1998)
Danio rerio	*ZDB-GENE-040801-49*	*nudcd2*	Q6DC89	4	157	CS (14-104); p23_NUDC_like (12-104)	cytoplasm	unfolded protein binding, protein folding	
Danio rerio	*ZDB-GENE-040426-2255*	*nudcd3*	A0A0R4IY34	6	344	CS (168-260); p23_NUDC_like (167-268)			
Danio rerio	*ZDB-GENE-040426-899*	*nudc*	Q6NV13	9	333	CS (169-260); p23_NUDC_like (174-260)			
Drosophila melanogaster	*FBgn0051251*	*DmelCG31251*	Q8IN95		306	CS (133-223)	cytoplasm, Golgi apparatus	unfolded protein binding, nuclear migration, protein folding	Miller et al. (2023); Wei et al. (2020); Hosono et al. (2015); Erives (2015); Cugusi et al. (2015); Neely et al. (2010); Schnorrer et al. (2010)
Drosophila melanogaster	*FBgn0021768*	*nudC*	Q9VVA6	6	332	CS (168-259); p23_NUDC_like (173-259)	cytoplasm, cytosol	unfolded protein binding, mRNA transport, nuclear migration, nucleus localization, positive regulation of dendrite morphogenesis, protein folding	Cunniff et al. (1997); Örkenby et al. (2023); Wainman et al. (2009)
Caenorhabditis elegans	*WBGene00003829*	*nud-1*	G5EE74	2	320	CS (156-247); p23_NUDC_like (161-247)	cytoplasm, synapse	identical protein binding, unfolded protein binding, chaperone-mediated protein folding, embryo development ending in birth or egg hatching, locomotion, pronuclear migration, protein folding, synaptic transmission, GABAergic, synaptic vesicle transport, vulval development	Faircloth et al. (2009); Dawe et al. (2001)
Schizosaccharomyces pombe	*SPBC19F8.02*	*nud3*	O60166	1	166	CS (6-97); p23_NUDC_like (11-97)	cytoplasm, microtubule, nucleus	unfolded protein binding, cell division, mitotic sister chromatid segregation, protein folding, protein maturation	
Dictyostelium discoideum	*DDB_G0286159*	*nudc*	Q54M64	2	171	CS (8-98); p23_NUDC_like (13-98)	cytoplasm, microtubule	unfolded protein binding, cell cycle, cell division, karyogamy, protein folding	

References

Aumais, J.P.; Tunstead, J.R.; McNeil, R.S.; Schaar, B.T.; McConnell, S.K.; Lin, S.H.; Clark, G.D.; Yu-Lee, L.Y. NudC associates with Lis1 and the dynein motor at the leading pole of neurons. *J. Neurosci.* **2001**, *21*, RC187; DOI: 10.1523/JNEUROSCI.21-24-j0002.2001

Aumais, J.P.; Williams, S.N.; Luo, W.; Nishino, M.; Caldwell, K.A.; Caldwell, G.A.; Lin, S.H.; Yu-Lee, L.Y. Role for NudC, a dynein-associated nuclear movement protein, in mitosis and cytokinesis. *J. Cell Sci.* **2003**, *116*, 1991-2003; DOI: 10.1242/jcs.00412

Bagci, H.; Sriskandarajah, N.; Robert, A.; Boulais, J.; Elkholi, I.E.; Tran, V.; Lin, Z.Y.; Thibault, M.P.; Dubé, N.; Faubert, D.; Hipfner, D.R. Mapping the proximity interaction network of the Rho-family GTPases reveals signalling pathways and regulatory mechanisms. *Nat. Cell Biol.* **2020**, *22*, 120-134; DOI: 10.1038/s41556-019-0438-7

Biebl, M.M.; Delhommel, F.; Faust, O.; Zak, K.M.; Agam, G.; Guo, X.; Mühlhofer, M.; Dahiya, V.; Hillebrand, D.; Popowicz, G.M.; Kampmann, M. NudC guides client transfer between the Hsp40/70 and Hsp90 chaperone systems. *Mol. Cell* **2022**, *82*, 555-569; DOI: 10.1016/j.molcel.2021.12.031

Burkard, T.R.; Planyavsky, M.; Kaupe, I.; Breitwieser, F.P.; Bürckstümmer, T.; Bennett, K.L.; Superti-Furga, G.; Colinge, J. Initial characterization of the human central proteome. *BMC Syst. Biol.* **2011**, *5*, 1-13; DOI: 10.1186/1752-0509-5-17

Cai, Y.; Yang, Y.; Shen, M.; Zhou, T. Inhibition of cytokinesis by overexpression of NudCL that is localized to the centrosome and midbody. *Cell Res.* **2009**, *19*, 1305-130; DOI: 10.1038/cr.2009.118

Chen, D.; Ito, S.; Yuan, H.; Hyodo, T.; Kadomatsu, K.; Hamaguchi, M.; Senga, T. EML4 promotes the loading of NUDC to the spindle for mitotic progression. *Cell Cycle* **2015**, *14*, 1529-1539; DOI: 10.1080/15384101.2015.1026514

Chen, W.; Wang, W.; Sun, X.; Xie, S.; Xu, X.; Liu, M.; Yang, C.; Li, M.; Zhang, W.; Liu, W.; Wang, L. NudCL2 regulates cell migration by stabilizing both myosin-9 and LIS1 with Hsp90. *Cell Death Dis.* **2020**, *11*, 534; DOI: 10.1038/s41419-020-02739-9

Chuang, C.; Pan, J.; Hawke, D.H.; Lin, S.H.; Yu-Lee, L.Y. NudC deacetylation regulates mitotic progression. *PloS one* **2013**, *8*, e73841; DOI: 10.1371/journal.pone.0073841

Cugusi, S.; Kallappagoudar, S.; Ling, H.; Lucchesi, J.C. The Drosophila Helicase Maleless (MLE) is Implicated in Functions Distinct From its Role in Dosage Compensation*[S]. *Mol. Cell. Proteomics* **2015**, *14*, 1478-1488; DOI: 10.1074/mcp.M114.040667

Cunniff, J.; Chiu, Y.H.; Morris, N.R.; Warrior, R. Characterization of DnudC, the Drosophila homolog of an Aspergillus gene that functions in nuclear motility. *Mech. Dev.* **1997**, *66*, 55-68; DOI: 10.1016/s0925-4773(97)00085-3

Dawe, A.L.; Caldwell, K.A.; Harris, P.M.; Morris, R.N.; Caldwell, G.A. Evolutionarily conserved nuclear migration genes required for early embryonic development in Caenorhabditis elegans. *Dev. Genes Evol.* **2001**, *211*, 434-441; DOI: 10.1007/s004270100176

De Souza, L.E.R.; Costa, M.D.M.; Bilek, E.S.; Lopes, M.H.; Martins, V.R.; Püschel, A.W.; Mercadante, A.F.; Nakao, L.S.; Zanata, S.M. STI1 antagonizes cytoskeleton collapse mediated by small GTPase Rnd1 and regulates neurite growth. *Exp. Cell Res.* **2014**, *324*, 84-91; DOI: 10.1016/j.yexcr.2014.03.017

Erives, A.J. Genes conserved in bilaterians but jointly lost with Myc during nematode evolution are enriched in cell proliferation and cell migration functions. *Dev. Genes Evol.* **2015**, *225*, 259-273; DOI: 10.1007/s00427-015-0508-1

Faircloth, L.M.; Churchill, P.F.; Caldwell, G.A.; Caldwell, K.A. The microtubule-associated protein, NUD-1, exhibits chaperone activity in vitro. *Cell Stress Chaperones* **2009**, *14*, 95-103; DOI: 10.1007/s12192-008-0061-1

Gladwyn-Ng, I.; Huang, L.; Ngo, L.; Li, S.S.; Qu, Z.; Vanyai, H.K.; Cullen, H.D.; Davis, J.M.; Heng, J.I.T. Bacurd1/Kctd13 and Bacurd2/Tnfaip1 are interacting partners to Rnd proteins which influence the long-term positioning and dendritic maturation of cerebral cortical neurons. *Neural Dev.* **2016**, *11*, 1-8; DOI: 10.1186/s13064-016-0062-1

Gladwyn-Ng, I.E.; Li, S.S.; Qu, Z.; Davis, J.M.; Ngo, L.; Haas, M.; Singer, J.; Heng, J.I.T. Bacurd2 is a novel interacting partner to Rnd2 which controls radial migration within the developing mammalian cerebral cortex. *Neural Dev.* **2015**, *10*, 1-13; DOI: 10.1186/s13064-015-0032-z

Hosono, C.; Matsuda, R.; Adryan, B.; Samakovlis, C. Transient junction anisotropies orient annular cell polarization in the Drosophila airway tubes. *Nat. Cell Biol.* **2015**, *17*, 1569-1576; DOI: 10.1038/ncb3267

Islam, M.A.; Choi, H.J.; Dash, R.; Sharif, S.R.; Oktaviani, D.F.; Seog, D.H.; Moon, I.S. N-acetyl-D-Glucosamine kinase interacts with NudC and Lis1 in dynein motor complex and promotes cell migration. *Int. J. Mol. Sci.* **2020**, *22*, 129; DOI: 10.3390/ijms22010129

Jurkuta, R.J.; Kaplinsky, N.J.; Spindel, J.E.; Barton, M.K. Partitioning the apical domain of the Arabidopsis embryo requires the BOBBER1 NudC domain protein. *Plant Cell* **2009**, *21*, 1957–1971; DOI: 10.1105/tpc.108.065284

Kaplinsky, N.J. Temperature compensation of auxin dependent developmental patterning. *Plant Signal. Behav*. **2009**, *4*, 12, 1157–1158; DOI: 10.4161/psb.4.12.9949

Li, M.; Xu, X.; Zhang, J.; Liu, M.; Wang, W.; Gao, Y.; Sun, Q.; Zhang, J.; Lu, Y.; Wang, F.; Liu, W. NudC-like protein 2 restrains centriole amplification by stabilizing HERC2. *Cell Death Dis.* **2019**, *10*, 628; DOI: 10.1038/s41419-019-1843-3

Lin, S.H.; Nishino, M.; Luo, W.; Aumais, J.P.; Galfione, M.; Kuang, J.; Yu-Lee, L.Y. Inhibition of prostate tumor growth by overexpression of NudC, a microtubule motor-associated protein. *Oncogene* **2004**, *23*, 2499-2506; DOI: 10.1038/sj.onc.1207343

Liu, S.; Wang, J.; Jiang, S.; Wang, H.; Gao, Y.; Zhang, H.; Li, D.; Song, F. Tomato SlSAP3, a member of the stress-associated protein family, is a positive regulator of immunity against *Pseudomonas syringae* pv. *tomato* DC3000. *Mol. Plant Pathol*. **2019**, *20*, 6, 815-830; DOI: 10.1111/mpp.12793

Matsumoto, N.; Ledbetter, D.H. Molecular cloning and characterization of the human NUDC gene. *Hum. Genet.* **1999**, *104*, 498-504; DOI: 10.1007/s004390050994

Miller, B.A.; Zhang, M.Y.; Gocke, C.D.; De Souza, C.; Osmani, A.H.; Lynch, C.; Davies, J.; Bell, L.; Osmani, S.A. A homolog of the fungal nuclear migration gene nudC is involved in normal and malignant human hematopoiesis. *Exp. Hematol.* **1999**, *27*, 742-750; DOI: 10.1016/s0301-472x(98)00074-5

Miller, D.E.; Dorador, A.P.; Van Vaerenberghe, K.; Li, A.; Grantham, E.K.; Cerbin, S.; Cummings, C.; Barragan, M.; Egidy, R.R.; Scott, A.R.; Hall, K.E. Off-target piRNA gene silencing in Drosophila melanogaster rescued by a transposable element insertion. *PLoS Genet.* **2023**, *19*, e1010598; DOI: 10.1371/journal.pgen.1010598

Morris, S.M.; Anaya, P.; Xiang, X.; Morris, N.R.; May, G.S.; Yu-Lee, L.Y. A prolactin-inducible T cell gene product is structurally similar to the Aspergillus nidulans nuclear movement protein NUDC. *Mol. Endocrinol.* **1997**, *11*, 229-236; DOI: 10.1210/mend.11.2.9892

Morris, S.M.; Yu-Lee, L.Y. Expression of RNUDC, a potential nuclear movement protein, in mammalian cells: localization to the Golgi apparatus. *Exp. Cell Res.* **1998**, *238*, 23-32; DOI: 10.1006/excr.1997.3822

Neely, G.G.; Kuba, K.; Cammarato, A.; Isobe, K.; Amann, S.; Zhang, L.; Murata, M.; Elmén, L.; Gupta, V.; Arora, S.; Sarangi, R. A global in vivo Drosophila RNAi screen identifies NOT3 as a conserved regulator of heart function. *Cell* **2010**, *141*, 142-153; DOI: 10.1016/j.cell.2010.02.023

Örkenby, L.; Skog, S.; Ekman, H.; Gozzo, A.; Kugelberg, U.; Ramesh, R.; Magadi, S.; Zambanini, G.; Nordin, A.; Cantú, C.; Nätt, D. Stress-sensitive dynamics of miRNAs and Elba1 in Drosophila embryogenesis. *Mol. Syst. Biol.* 2023, *19*, e11148; DOI: 10.15252/msb.202211148

Pang, S.F.; Li, X.K.; Zhang, Q.; Yang, F.; Xu, P. Interference RNA (RNAi)-based silencing of endogenous thrombopoietin receptor (Mpl) in Dami cells resulted in decreased hNUDC-mediated megakaryocyte proliferation and differentiation. *Exp. Cell Res.* **2009**, *315*, 3563-3573; DOI: 10.1016/j.yexcr.2009.06.020

Perez, D.E.; Hoyer, J.S.; Johnson, A.I.; Moody, Z.R.; Lopez, J.; Kaplinsky, N.J. *BOBBER1* is a noncanonical Arabidopsis small heat shock protein required for both development and thermotolerance. *Plant Physiol*. **2009**, *151*, 241–252; DOI: 10.1104/pp.109.142125

Rose, J.E.; Behm, F.M.; Drgon, T.; Johnson, C.; Uhl, G.R. Personalized smoking cessation: interactions between nicotine dose, dependence and quit-success genotype score. *Mol. Med.* **2010**, *16*, 247-253; DOI: 10.2119/molmed.2009.00159

Scheuerle, A.E.; Ni, M.; Ahmad, A.A.; Timmons, C.F.; Rakheja, D.; Gordon, E.E.; Boothe, M. Biallelic variants in NUDCD2 associated with a multiple malformation syndrome with cholestasis and renal failure. *Am. J. Med. Genet. A* **2023**, *191*, 2324-2328; DOI: 10.1002/ajmg.a.63314.

Schnorrer, F.; Schönbauer, C.; Langer, C.C.; Dietzl, G.; Novatchkova, M.; Schernhuber, K.; Fellner, M.; Azaryan, A.; Radolf, M.; Stark, A.; Keleman, K. Systematic genetic analysis of muscle morphogenesis and function in Drosophila. *Nature* **2010**, *464*, 287-291; DOI: 10.1038/nature08799

Skarnes, W.C.; Rosen, B.; West, A.P.; Koutsourakis, M.; Bushell, W.; Iyer, V.; Mujica, A.O.; Thomas, M.; Harrow, J.; Cox, T., Jackson, D. A conditional knockout resource for the genome-wide study of mouse gene function. *Nature* **2011**, *474*, 337-342; DOI: 10.1038/nature10163

Tang, Y.S.; Zhang, Y.P.; Xu, P. hNUDC promotes the cell proliferation and differentiation in a leukemic cell line via activation of the thrombopoietin receptor (Mpl). *Leukemia* **2008**, *22*, 1018-1025; DOI: 10.1038/leu.2008.20

Velinov, V.; Vaseva, I.; Zehirov, G.; Zhiponova, M.; Georgieva, M.; Vangheluwe, N.; Beeckman, T.; Vassileva, V. Overexpression of the *NMig1* gene encoding a NudC domain protein enhances root growth and abiotic stress tolerance in *Arabidopsis thaliana*. *Front. Plant Sci*. **2020**, *11*, 815; DOI: 10.3389/fpls.2020.00815

Velinov, V.; Georgieva, M.; Zehirov, G.; Vassileva, V. *NudC-like* genes contribute to root growth and branching in *Arabidopsis thaliana*. *C. R. Acad. Bulg. Sci*. **2021**, *74*, 12, 1767-1773; DOI: 10.7546/CRABS.2021.12.06

Wainman, A.; Creque, J.; Williams, B.; Williams, E.V.; Bonaccorsi, S.; Gatti, M.; Goldberg, M.L. Roles of the Drosophila NudE protein in kinetochore function and centrosome migration. *J. Cell Sci.* **2009**, *122*, 1747-1758 DOI: 10.1242/jcs.041798

Wei, P.; Xue, W.; Zhao, Y.; Ning, G.; Wang, J. CRISPR-based modular assembly of a UAS-cDNA/ORF plasmid library for more than 5500 Drosophila genes conserved in humans. *Genome Res.* **2020**, *30*, 95-106; DOI: 10.1101/gr.250811.119

Weiderhold, K.N.; Fadri-Moskwik, M.; Pan, J.; Nishino, M.; Chuang, C.; Deeraksa, A.; Lin, S.H.; Yu-Lee, L.Y. Dynamic phosphorylation of NudC by Aurora B in cytokinesis. *PLoS One* **2016**, *11*, e0153455; DOI: 10.1371/journal.pone.0153455

Yamada, M.; Toba, S.; Takitoh, T.; Yoshida, Y.; Mori, D.; Nakamura, T.; Iwane, A.H.; Yanagida, T.; Imai, H.; Yu-Lee, L.Y.; Schroer, T. mNUDC is required for plus-end-directed transport of cytoplasmic dynein and dynactins by kinesin-1. *Embo J* **2010**, *29*, 517-531; DOI: 10.1038/emboj.2009.378

Yang, J.; Zhang, L.; Li, Y.; Chen, M. Identifying key m6A-methylated lncRNAs and genes associated with neural tube defects via integrative MeRIP and RNA sequencing analyses. *Front. Genet.* **2022**, *13*, 974357; DOI: 10.3389/fgene.2022.974357

Zhou, L.; Di, Q.; Sun, B.; Wang, X.; Li, M.; Shi, J. MicroRNA-194 restrains the cell progression of non-small cell lung cancer by targeting human nuclear distribution protein C. *Oncol. Rep.* **2016**, *35*, 3435-3444; DOI: 10.3892/or.2016.4708

Zhou, T.; Aumais, J.P.; Liu, X.; Yu-Lee, L.Y.; Erikson, R.L. A role for Plk1 phosphorylation of NudC in cytokinesis. *Dev. Cell* **2003**, *5*, 127-138; DOI: 10.1016/s1534-5807(03)00186-2

Zhou, T.; Zimmerman, W.; Liu, X.; Erikson, R.L. A mammalian NudC-like protein essential for dynein stability and cell viability. *Proc. Natl. Acad. Sci. U. S. A.* **2006**, *103*, 9039-9044; DOI: 10.1073/pnas.0602916103

Zhu, X.J.; Liu, X.; Jin, Q.; Cai, Y.; Yang, Y.; Zhou, T. The L279P mutation of nuclear distribution gene C (NudC) influences its chaperone activity and lissencephaly protein 1 (LIS1) stability. *J. Biol. Chem.* **2010**, *285*, 29903-29910; DOI: 10.1074/jbc.M110.105494
